# Double lock of a potent human therapeutic monoclonal antibody against SARS-CoV-2

**DOI:** 10.1093/nsr/nwaa297

**Published:** 2020-12-18

**Authors:** Ling Zhu, Yong-Qiang Deng, Rong-Rong Zhang, Zhen Cui, Chun-Yun Sun, Chang-Fa Fan, Xiaorui Xing, Weijin Huang, Qi Chen, Na-Na Zhang, Qing Ye, Tian-Shu Cao, Nan Wang, Lei Wang, Lei Cao, Huiyu Wang, Desheng Kong, Juan Ma, Chunxia Luo, Yanjing Zhang, Jianhui Nie, Yao Sun, Zhe Lv, Neil Shaw, Qianqian Li, Xiao-Feng Li, Junjie Hu, Liangzhi Xie, Zihe Rao, Youchun Wang, Xiangxi Wang, Cheng-Feng Qin

**Affiliations:** CAS Key Laboratory of Infection and Immunity, National Laboratory of Macromolecules, Institute of Biophysics, Chinese Academy of Sciences, Beijing 100101, China; State Key Laboratory of Pathogen and Biosecurity, Beijing Institute of Microbiology and Epidemiology, Academy of Military Medical Sciences, Beijing 100071, China; State Key Laboratory of Pathogen and Biosecurity, Beijing Institute of Microbiology and Epidemiology, Academy of Military Medical Sciences, Beijing 100071, China; CAS Key Laboratory of Infection and Immunity, National Laboratory of Macromolecules, Institute of Biophysics, Chinese Academy of Sciences, Beijing 100101, China; Beijing Engineering Research Center of Protein and Antibody, Sinocelltech Ltd., Beijing 100176, China; Division of Animal Model Research, Institute for Laboratory Animal Resources, National Institutes for Food and Drug Control (NIFDC), Beijing 102629, China; CAS Key Laboratory of Infection and Immunity, National Laboratory of Macromolecules, Institute of Biophysics, Chinese Academy of Sciences, Beijing 100101, China; School of Basic Medical Sciences, Southwest Medical University, Luzhou 646000, China; Division of HIV/AIDS and Sex-Transmitted Virus Vaccines, Institute for Biological Product Control, NIFDC, Beijing 102629, China; State Key Laboratory of Pathogen and Biosecurity, Beijing Institute of Microbiology and Epidemiology, Academy of Military Medical Sciences, Beijing 100071, China; State Key Laboratory of Pathogen and Biosecurity, Beijing Institute of Microbiology and Epidemiology, Academy of Military Medical Sciences, Beijing 100071, China; State Key Laboratory of Pathogen and Biosecurity, Beijing Institute of Microbiology and Epidemiology, Academy of Military Medical Sciences, Beijing 100071, China; State Key Laboratory of Pathogen and Biosecurity, Beijing Institute of Microbiology and Epidemiology, Academy of Military Medical Sciences, Beijing 100071, China; CAS Key Laboratory of Infection and Immunity, National Laboratory of Macromolecules, Institute of Biophysics, Chinese Academy of Sciences, Beijing 100101, China; CAS Key Laboratory of Infection and Immunity, National Laboratory of Macromolecules, Institute of Biophysics, Chinese Academy of Sciences, Beijing 100101, China; CAS Key Laboratory of Infection and Immunity, National Laboratory of Macromolecules, Institute of Biophysics, Chinese Academy of Sciences, Beijing 100101, China; Beijing Engineering Research Center of Protein and Antibody, Sinocelltech Ltd., Beijing 100176, China; Beijing Engineering Research Center of Protein and Antibody, Sinocelltech Ltd., Beijing 100176, China; Beijing Engineering Research Center of Protein and Antibody, Sinocelltech Ltd., Beijing 100176, China; Beijing Engineering Research Center of Protein and Antibody, Sinocelltech Ltd., Beijing 100176, China; Beijing Engineering Research Center of Protein and Antibody, Sinocelltech Ltd., Beijing 100176, China; Division of HIV/AIDS and Sex-Transmitted Virus Vaccines, Institute for Biological Product Control, NIFDC, Beijing 102629, China; CAS Key Laboratory of Infection and Immunity, National Laboratory of Macromolecules, Institute of Biophysics, Chinese Academy of Sciences, Beijing 100101, China; CAS Key Laboratory of Infection and Immunity, National Laboratory of Macromolecules, Institute of Biophysics, Chinese Academy of Sciences, Beijing 100101, China; CAS Key Laboratory of Infection and Immunity, National Laboratory of Macromolecules, Institute of Biophysics, Chinese Academy of Sciences, Beijing 100101, China; Division of HIV/AIDS and Sex-Transmitted Virus Vaccines, Institute for Biological Product Control, NIFDC, Beijing 102629, China; State Key Laboratory of Pathogen and Biosecurity, Beijing Institute of Microbiology and Epidemiology, Academy of Military Medical Sciences, Beijing 100071, China; CAS Key Laboratory of Infection and Immunity, National Laboratory of Macromolecules, Institute of Biophysics, Chinese Academy of Sciences, Beijing 100101, China; Beijing Engineering Research Center of Protein and Antibody, Sinocelltech Ltd., Beijing 100176, China; Beijing Key Laboratory of Monoclonal Antibody Research and Development, Sino Biological Inc., Beijing 100176, China; Cell Culture Engineering Center, Chinese Academy of Medical Sciences & Peking Union Medical College, Beijing 100005, China; CAS Key Laboratory of Infection and Immunity, National Laboratory of Macromolecules, Institute of Biophysics, Chinese Academy of Sciences, Beijing 100101, China; Division of HIV/AIDS and Sex-Transmitted Virus Vaccines, Institute for Biological Product Control, NIFDC, Beijing 102629, China; CAS Key Laboratory of Infection and Immunity, National Laboratory of Macromolecules, Institute of Biophysics, Chinese Academy of Sciences, Beijing 100101, China; Guangzhou Regenerative Medicine and Health Guangdong Laboratory, Guangzhou 510200, China; State Key Laboratory of Pathogen and Biosecurity, Beijing Institute of Microbiology and Epidemiology, Academy of Military Medical Sciences, Beijing 100071, China

**Keywords:** SARS-CoV-2, COVID-19, *in vivo* protection, preclinical safety evaluation, human neutralizing antibody, immuno-therapy, cryo-EM structure

## Abstract

Receptor recognition and subsequent membrane fusion are essential for the establishment of successful infection by SARS-CoV-2. Halting these steps can cure COVID-19. Here we have identified and characterized a potent human monoclonal antibody, HB27, that blocks SARS-CoV-2 attachment to its cellular receptor at sub-nM concentrations. Remarkably, HB27 can also prevent SARS-CoV-2 membrane fusion. Consequently, a single dose of HB27 conferred effective protection against SARS-CoV-2 in two established mouse models. Rhesus macaques showed no obvious adverse events when administrated with 10 times the effective dose of HB27. Cryo-EM studies on complex of SARS-CoV-2 trimeric S with HB27 Fab reveal that three Fab fragments work synergistically to occlude SARS-CoV-2 from binding to the ACE2 receptor. Binding of the antibody also restrains any further conformational changes of the receptor binding domain, possibly interfering with progression from the prefusion to the postfusion stage. These results suggest that HB27 is a promising candidate for immuno-therapies against COVID-19.

## INTRODUCTION

On 11 March 2020, the World Health Organization declared the 2019 coronavirus disease (COVID-19) a pandemic. Severe acute respiratory syndrome coronavirus 2 (SARS-CoV-2), the etiological agent of this pandemic, continues to ravage the global population, causing millions of infections. Losses of lives, declining wellbeing and disruption of economic activities as a result of the infections have strained societies and significantly impacted upon people's normal lives. SARS-CoV-2 belongs to the betacoronavirus genus, five coronaviruses of which, together with two alphacoronaviruses, are endowed with an ability to infect humans [1,2]. Among these, infections caused by SARS-CoV, SARS-CoV-2 and Middle East Respiratory Syndrome coronavirus (MERS-CoV) are known to culminate in more severe clinical manifestations [3]. To date, no specific drugs or vaccines effective against these highly pathogenic coronaviruses have been approved.

Like SARS-CoV, SARS-CoV-2 utilizes its protuberant S glycoprotein to engage with its cellular receptor, human angiotensin converting enzyme 2 (ACE2), for forging membrane fusion in order to enter the host cell [4,5]. Each monomeric S protein can be cleaved by host proteases, such as TMPRSS2 [5,6], into two functional domains, the distal globular S1 domain and the membrane-proximal S2 domain, which mediate receptor binding and membrane fusion, respectively [7]. The S1 subunit consists of an N-terminal domain (NTD) and a C-terminal domain, which often functions as the receptor binding domain (RBD). Conformational transitions are triggered upon release of the S1 subunit after receptor binding and subsequent priming of the protein by host cell proteases. These two key events advance the life cycle of the virus from the prefusion to the postfusion stage, leading to the fusion of the viral membrane with that of the host cell [7,8].

Such important roles played by S during viral infection make them valuable targets for antibody-based drug and vaccine design [9]. Previous structural studies have revealed that the S trimer can switch between a receptor-accessible state where one or more RBDs are in the open conformation and a receptor-inaccessible state where all the RBDs are in the closed conformation. This switch is accomplished through a hinge-like movement of the RBD, indicative of a dynamic and complicated protein-protein interaction mode with host cells [10–14]. Although numerous neutralizing antibodies (NAbs) targeting the RBDs of SARS-CoV or MERS have been reported [15–17], the immunogenic features and key epitopes of SARS-CoV-2 remain poorly characterized. Recently, a cross-binding monoclonal antibody (mAb), CR3022, was demonstrated to neutralize SARS-CoV, but it failed to efficiently prevent SARS-CoV-2 infection, highlighting the challenges posed by conformationally flexible virus-specific neutralizing epitopes in conferring protection against infection [18]. More recently, a number of NAbs have been shown to block the binding of SARS-CoV-2 to ACE2 and another RBD-targeting NAb, S309, acted by inducing antibody-dependent cell cytotoxicity (ADCC) which surprisingly did not involve the blocking of virus-receptor interaction [19–28]. This raises the possibility of the existence of hitherto undiscovered neutralization mechanisms for SARS-CoV-2 RBD-targeting NAbs. A detailed understanding of the mechanisms underlying the neutralization of SARS-CoV-2 is likely to help provide new guidance for the development of antiviral therapeutics and rational vaccine design.

## RESULTS

### Phage display identifies a potent SARS-CoV-2-specific NAb

**Figure 1.**
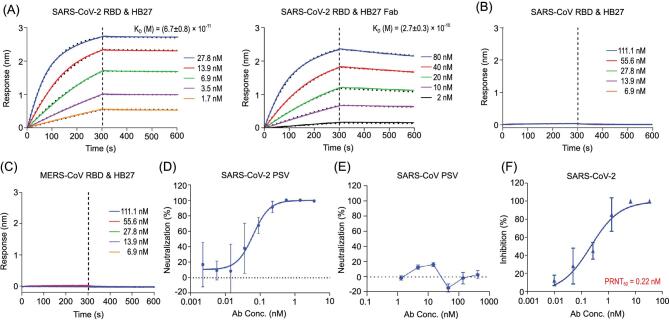
HB27 is a SARS-CoV-2-specific antibody of high potency. (A) Analysis of affinity of HB27 (left panel) and HB27 Fab fragments (right panel) for SARS-CoV-2 RBD. Biotinylated SARS-CoV-2 RBD protein was loaded on Octet SA sensor and tested for real-time association and dissociation from HB27 IgG and HB27 Fab fragments, respectively. (B) and (C) Analysis of affinity of HB27 for SARS-CoV RBD and MERS-CoV RBD, respectively. (D) and (E) Neutralizing activity of HB27 against SARS-CoV-2 and SARS-CoV pseudoviruses (PSV), respectively. Serially diluted HB27 titers were added to test neutralizing activity against SARS-CoV-2 and SARS-CoV PSV. (F) *In vitro* neutralization activity of HB27 against SARS-CoV-2 by plaque reduction neutralization test (PRNT) in Vero cells. Neutralizing activities are represented as mean ± SD. Experiments were performed in duplicates.

We previously identified a set of NAbs from an antibody library which was generated from RNAs extracted from peripheral lymphocytes of mice immunized with recombinant SARS-CoV RBD protein [29]. In this study, we constructed another antibody library by immunizing mice with recombinant SARS-CoV-2 RBD, which yielded a chimeric anti-SARS-CoV-2 mAb, named mhB27. mhB27 was able to strongly bind to SARS-CoV-2 RBD and exhibited potent neutralizing activities against SARS-CoV-2 when tested in a vesicular stomatitis virus (VSV) pseudotyping system (PSV) (Supplementary Fig. S1). A humanized antibody HB27 was generated based on the sequences of mhB27. To investigate the viral specificity of HB27, we performed binding assays measuring the ability of HB27 to bind the RBDs of SARS-CoV, SARS-CoV-2 and MERS-CoV. Analysis of the data obtained from real-time quantitation and kinetic characterization of biomolecular interactions using OCTET system demonstrated that both immunoglobulin G (IgG) and Fab fragments of HB27 bind tightly to SARS-CoV-2 RBD with affinities of 0.07 nM and 0.27 nM, respectively. However, this antibody exhibits undetectable interactions with the RBDs of SARS-CoV and MERS-CoV, suggesting that HB27 is SARS-CoV-2-specific (Fig. 1A–C). HB27 showed potent neutralizing activities against SARS-CoV-2 with a 50% inhibition concentration (IC_50_) value of 0.04 nM. Perhaps correlated with the inability to interact with SARS-CoV RBD, HB27 possessed no inhibition activity against SARS-CoV in PSV-based neutralization assays (Fig. 1D and E). Classical plaque reduction neutralization test (PRNT) conducted against an authentic SARS-CoV-2 strain (BetaCoV/Beijing/IME-BJ01/2020) further verified its neutralizing activity with a PRNT_50_ value of 0.22 nM (Fig. 1F).

### Prophylactic and therapeutic efficacy of HB27 in SARS-CoV-2 susceptible mice

**Figure 2.**
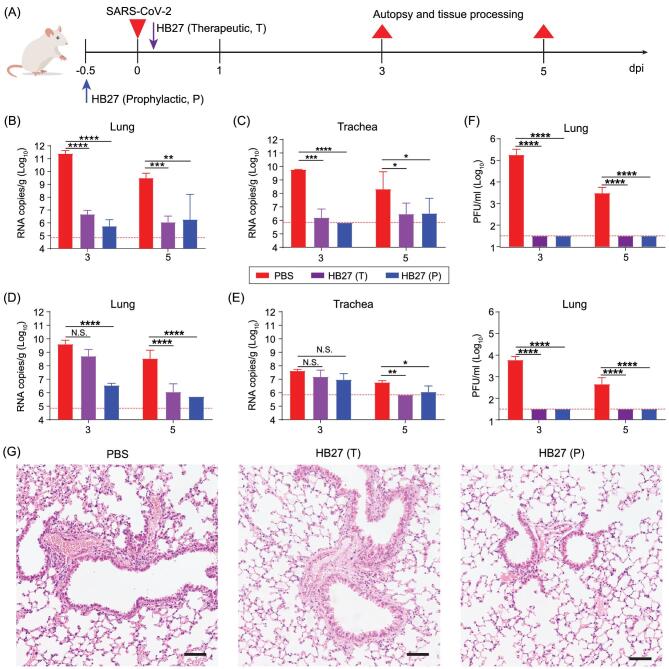
Prophylactic and therapeutic efficacy of HB27 in two SARS-CoV-2 susceptible mice models. (A) Experimental design for therapeutic and prophylactic evaluations of HB27 in two SARS-CoV-2 susceptible mice models. Group of 6-to-8-week-old hACE2 mice and BALB/c mice were infected intranasally with 5 × 10^4^ PFU of SARS-CoV-2 BetaCoV/Beijing/IME-BJ01/2020 or 1.6 × 10^4^ PFU of MASCp6 as described previously, respectively. A dose of 20 mg/kg HB27 was injected intraperitoneally at 12 h before infection (the prophylactic group, P) or at 2 h after infection (the therapeutic group, T). PBS (phosphate buffered saline) injections were used as control group. Then, the lung tissues of mice were collected at 3 and 5 dpi for virus titer, H&E and immunostaining. (B) and (C) Virus titers of lung and trachea tissues at 3 or 5 dpi in mouse model based on a SARS-CoV-2 mouse adapted strain MASCp6. The viral loads of the tissues were determined by qRT-PCR (^*^P < 0.05; ^**^P < 0.01; ^***^P < 0.001; ^****^P < 0.0001; N.S., not significant). Data are represented as mean ± SD. Dashed lines represent limit of detection. (D) and (E) Virus titers of lung and trachea tissues at 3 or 5 dpi in hACE2 humanized mouse model. The viral loads of the tissues were determined by qRT-PCR (^*^P < 0.05; ^**^P < 0.01; ^***^P < 0.001; ^****^P < 0.0001; N.S., not significant). Data are represented as mean ± SD. Dashed lines represent limit of detection. (F) Viral burden at 3 or 5 dpi in the lungs from two mouse models (top: BALB/c mice; bottom: hACE2 mice), measured by plaque assay. Data are represented as mean ± SD. Dashed lines represent limit of detection. (G) Histopathological analysis of lung samples at 5 dpi. Scale bar: 100 μm.

Given the excellent neutralizing activities at sub-nM concentrations, we next sought to assess the correlation between *in vitro* neutralization and *in vivo* protection. The HB27 produced in the CHO cell line was first tested in a newly established mouse model based on a SARS-CoV-2 mouse adapted strain MASCp6 [30]. Upon MASCp6 intranasal challenge, adult BALB/c sustained robust viral replication in the lungs at 3–5 days post inoculation. To evaluate the protection efficacy of HB27, BALB/c mice challenged with MAScp6 were administered a single dose of 20 mg/kg of HB27 in prophylactic as well as therapeutic settings (Fig. 2A). As expected, high levels of viral RNAs were detected in the lungs and trachea at 3 and 5 days post-infection in the control group of mice treated with phosphate buffered saline (PBS) (Fig. 2B and C). Remarkably, a single dose of HB27 administered either before or post-SARS-CoV-2 exposure resulted in >99.9% reduction of the viral RNA loads in the lungs and trachea (Fig. 2B and C).

Furthermore, we validated the *in vivo* protection efficacy of HB27 in a human ACE2 (hACE2) humanized mouse model that was susceptible to SARS-CoV-2 infection [31]. Similar to the studies with the MASCp6 strain of mice, either prophylactic or therapeutic administration of HB27 conferred a clear benefit on the hACE2 humanized mouse model as indicated by a significant reduction in viral RNA loads in the lungs and trachea at day five post-SARS-CoV-2 challenge. Prophylactic administration of HB27 showed a more potent antiviral effect, resulting in >1000- fold reduction in lung viral levels (Fig. 2D and E). A direct challenge via administration of excessive (up to 5 × 10^5^ PFU (Plaque forming units)) SARS-CoV-2 through the intranasal route, where the IgG antibodies may not be able to directly engage the target, could lead to virus particles gaining access to the lung and trachea. Such experimental observations in the prophylactic and therapeutic settings for many other protective human antibodies against SARS-CoV-2 have been reported [29,32]. However, it is worthy of note that no infectious virions could be detected in the lung at day three and day five as measured by a plaque assay of lung tissue homogenates (Fig. 2F). These results suggest that the low levels of viral RNA copies detected in the lung/trachea might be the remnants of the viral genomes from the infection at the very early stage. Histopathological examination revealed moderate interstitial pneumonia, characterized by inflammatory cell infiltration, alveolar septal thickening and distinctive vascular system injury developed in hACE2 humanized mice belonging to the PBS control group at day five (Fig. 2G). In contrast, the lungs in mice from the HB27 treated group only showed minimal or very mild inflammatory cell infiltration, and no obvious lesions of alveolar epithelial cells or focal hemorrhage (Fig. 2G). Collectively, these results clearly demonstrated the utility of HB27 for prophylactic or therapeutic purposes against SARS-CoV-2.

### Evaluation of the safety of administration of HB27 in non-human primates

**Figure 3.**
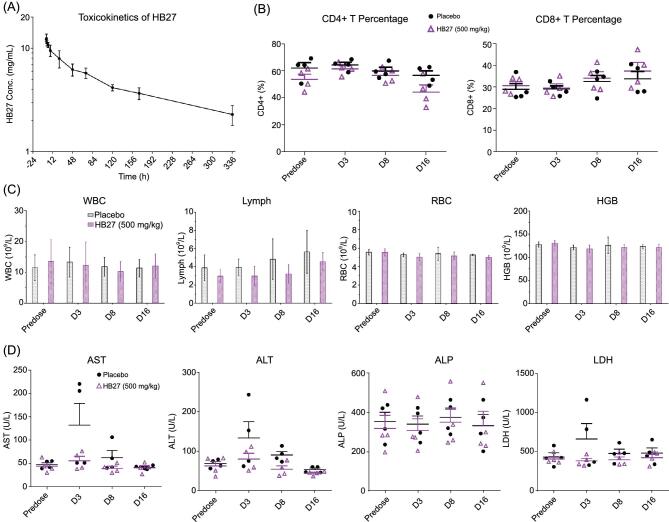
Safety evaluation of HB27 in rhesus macaques. (A) The toxicokinetics of HB27 in rhesus macaques was evaluated by measuring HB27 levels in serum predose and at 12, 48, 84, 120, 156, 192, 228, 264, 300 and 336 h after administration. (B–D) Rhesus macaques were given intravenous injections of a single dose of either placebo or HB27 (500 mg/kg), and monitored by (B) lymphocyte subset analysis, (C) hematological test, and (D) biochemical blood test predose, and 3, 8 and 16 days postdose. WBC: white blood cells; Lymph: lymphocytes; RBC: red blood cells; HBG: hemoglobin; AST: aspartate transaminase; ALT: alanine transaminase; ALP: alkaline phosphatase; LDH: lactate dehydrogenase.

As part of the non-clinical safety studies prior to the conduction of human clinical trials for pharmaceuticals, we systematically evaluated the safety of administration of HB27 in rhesus macaques. Two groups of four animals (*n* = 4) were administered a single intravenous high dose (500 mg/kg, 10 times the estimated effective dose in humans) of HB27 or placebo. HB27 serum concentrations, clinical observations and biological indices were monitored closely for 16 days (Fig. 3). Neither fever nor weight loss was observed in any macaque after the infusion of HB27, and the appetite and mental state of all animals remained normal. The toxicokinetics of HB27 in rhesus macaques was evaluated by measuring HB27 levels in serum pre-infusion and at indicated time intervals after administration. A mean maximum serum concentration (C_max_) of 12.8 mg/mL (± 0.8) of HB27 could be achieved and the average half-life of the antibody was 10.0 days (± 2.2) (Fig. 3A and Supplementary Table S1). Notably, prophylactic and therapeutic efficacy of HB27 in animal models revealed that >99.9% of the viral RNA loads in the lungs and trachea could be reduced at 5 days post-infection (Fig. 2), suggesting that a half-life of 10 days for HB27 is probably sufficient for deriving therapeutic benefit. Details of the results of the measurements of toxicokinetic parameters are presented in Supplementary Table S1. These results suggest that HB27 probably has pharmacokinetic properties consistent with a typical human IgG1. Hematological and biochemical analysis, including biochemical blood tests and lymphocyte subset percent (CD4^+^ and CD8^+^) showed no notable changes in the HB27 administrated group when compared to the placebo group (Fig. 3B and D). Taken together, the results of our animal studies indicate that HB27 is generally safe in non-human primates.

### HB27 prevents the attachment of SARS-CoV-2 to host cells by blocking its binding to ACE2

**Figure 4.**
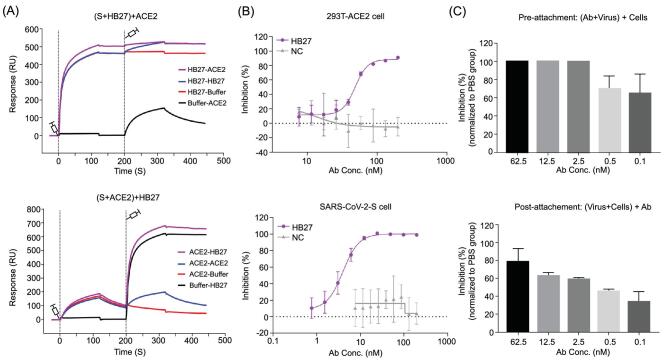
HB27 blocks the interactions of SARS-CoV-2 with ACE2. (A) BIAcore SPR kinetics showing the competitive binding of HB27 and ACE2 to SARS-CoV-2 S trimer. For both panels, SARS-CoV-2 S protein was immobilized onto the sensor chips. In the upper panel, HB27 was first injected, followed by ACE2, whereas in the lower panel, ACE2 was injected first and then HB27. The control groups are as shown by the curves. (B) Blocking of SARS-CoV-2 RBD binding to 293T-ACE2 cells by HB27 (upper panel). Recombinant SARS-CoV-2 RBD protein and serially diluted HB27 were incubated with ACE2 expressing 293T cells (293T-ACE2) and tested for binding of HB27 to 293T-ACE2 cells. Competitive binding of HB27 and ACE2 to SARS-CoV-2-S cells (lower panel). Recombinant ACE2 and serially diluted HB27 were incubated with 293T cells expressing SARS-CoV-2 S (SARS-CoV-2-S) and tested for binding of HB27 to SARS-CoV-2-S cells. BSA was used as a negative control (NC). (C) Amount of virus on the cell surface, as detected by RT-PCR, when exposed to HB27 prior to (upper panel) and after (lower panel) the virus was allowed to attach to cells. Values are mean ± SD. Experiments were repeated in triplicate.

To evaluate the ability of HB27 to inhibit binding of RBD to ACE2, we performed competitive binding assays at both protein and cellular levels. Results of the enzyme-linked immunosorbent assay (ELISA) revealed that HB27 could prevent the binding of soluble ACE2 (monomer in solution) to SARS-CoV-2 RBD with an EC_50_ value of 0.5 nM (Supplementary Fig. S2A). To verify the ability of HB27 to block the binding of ACE2 to trimeric S, we expressed and purified stabilized SARS-CoV-2 S ectodomain trimer. Surface plasmon resonance (SPR) assays indicated that HB27 interacts with SARS-CoV-2 S trimer with a slightly stronger binding affinity (∼0.04 nM) (Supplementary Fig. S2B), which was ∼1000 times higher than that of soluble ACE2 with SARS-CoV-2 S (Supplementary Fig. S2C) [6]. For the competitive SPR, two sets of assays: exposing the trimeric S to HB27 first and then to soluble ACE2, or the other way around, were conducted. As expected, binding of HB27 completely blocked the interaction between soluble ACE2 and SARS-CoV-2 trimeric S. Moreover, soluble ACE2 that had already bound to trimeric S could be replaced by HB27 because of the ∼1000-fold difference in binding affinities of these ligands to the SARS-CoV-2 trimeric S (Fig. 4A). Cell-based immunofluorescent blocking assays demonstrated that HB27 could block both the binding of soluble ACE2 to SARS-CoV-2 S expressing 293T cells and the attachment of SARS-CoV-2 RBD to ACE2 expressing 293T cells in a dose-dependent manner albeit with relative high EC_50_ values of ∼5–50 nM (Fig. 4B and Supplementary Fig. S2D). Overexpression of ACE2/S trimer on the 293T cell surface and the presence of the dimeric form of ACE2 on the cell surface are probably the reasons for the substantially higher concentration of HB27 needed to prevent attachment of the virus to the cell surface. To further verify these results in the cell-based viral infection model, we used real-time reverse transcriptase–PCR (RT–PCR) to quantify the amount of virus remaining on the surface of cells that were treated with HB27 pre- and post-viral attachment at 4°C. In line with the results of the competitive binding assays, HB27 efficiently prevented SARS-CoV-2 attachment to the host cell surface at sub-nM and could displace the virions that had already bound to the cell surface at ∼2.5 nM (Fig. 4C).

### HB27 prevents SARS-CoV-2 membrane fusion

**Figure 5.**
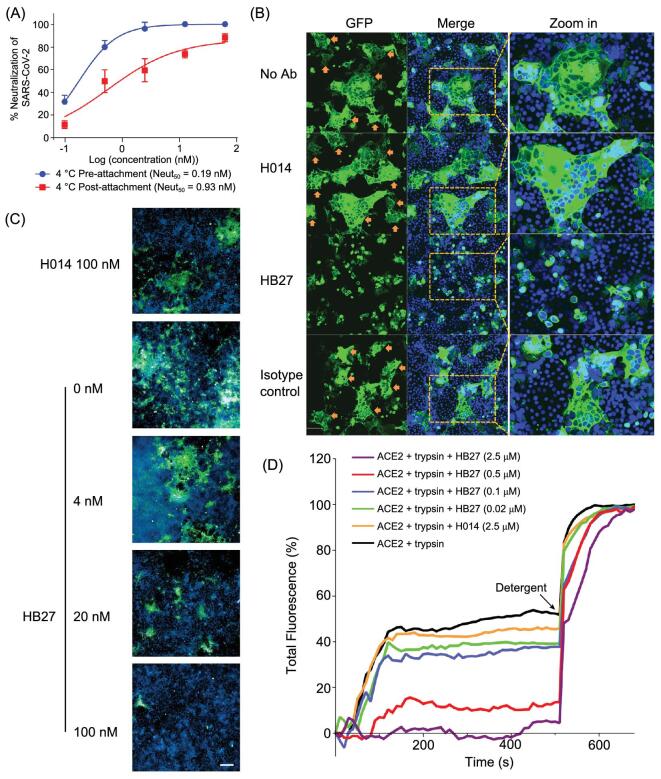
HB27 inhibits SARS-CoV-2 membrane fusion. (A) HB27 had potent neutralization activities when exposed to virus before or after attachment to Huh7 cells. Values are mean ± SD. Experiments were repeated in triplicate. (B) HB27 inhibits S protein-mediated cell–cell fusion. 293T cells were transfected with SARS-CoV-2 S-GFP protein, co-cultured with Vero E6 cells in the absence or presence of 100 μg/mL H014 or HB27 or anti-influenza H7N9 antibody (isotype control). No Ab: in the absence of antibodies. Images were taken after 48 h. Cells were fixed with 4% paraformaldehyde (PFA) at room temperature for 20 min and stained for nuclei with 4,6-diamidino-2-phenylindole (DAPI). (C) HB27 inhibits SARS-CoV-2-mediated cell–cell fusion. Huh7 cells were infected with 100 PFU of SARS-CoV-2 for 1 h at 4°C and washed three times. After that, cells were further cultured in the presence of a series of concentrations (0, 4, 20 and 100 nM) of HB27, or 100 nM of H014 at 37°C for 48 h. Images were taken after 48 h. Cells were fixed with 4% (w/v) PFA for 20 min and incubated with anti-SARS-CoV-2 S protein antibody and stained for nuclei with DAPI. Scale bar equals 200 μm. (D) HB27 blocks receptor-mediated fusion of SARS-CoV-2 with liposomes. Liposomes were loaded with self-quenching concentrations of the fluorescent dye calcein. Perturbation of the bilayer causes the release of calcein resulting in dilution and a consequent increase in its fluorescence. Fusion of SARS-CoV-2 with liposomes occurred in the presence of both ACE2 and trypsin and a series of HB27 concentrations were used to inhibit the fusion. 10% Triton X-100 treatment was used to achieve 100% calcein leakage. All data shown are representative of three independent experiments.

A common way to determine whether the antibody inhibits virus-receptor binding or a post-attachment step of the infection is to compare neutralization curves deduced from mixing antibodies with the virus before or after binding to cells at 4°C. The assumption is that antibodies that inhibit receptor binding will not have a neutralizing effect on virus that has already bound to its receptor. However, this assumption may not be true, because high-affinity antibodies could possibly displace the virus that is already complexed to a low-affinity receptor, as observed for HB27 (Fig. 4B and C). Thus, deriving the mechanism of neutralization by just conducting neutralization assays may not yield the complete picture, although pre- and post-attachment neutralization assays suggested that HB27 inhibits a post-attachment step of the infection (Fig. 5A). In coronaviruses, receptor binding and proteolytic processing act in synergy to trigger a series of conformational changes in S, bringing viral and cellular membranes in proximity for fusion, leading to establishment of infection [8]. TMPRSS2-mediated cleavage is capable of activating the fusion potential of coronavirus S proteins, inducing receptor-dependent syncytium formation, which was recently observed in natural SARS-CoV-2 infections as well [33,34]. To explore whether HB27 could interfere with syncytium formation, we established the S-mediated cell–cell fusion system using 293T cells that express SARS-CoV-2 S with a Green fluorescent protein (GFP) tag as the effector cells and Vero-E6 cells as the target cells (Fig. 5B). After co-incubation of effector and target cells for 48 h, hundreds of cells fused together into one large syncytium with multiple nuclei (Fig. 5B). Remarkably, HB27 could completely inhibit SARS-CoV-2 mediated cell–cell fusion at the concentration of 0.5 μM. Notably, this result is comparable with the inhibition efficiencies of some pan-coronavirus fusion inhibitors (Fig. 5B) [34]. Neither SARS-CoV-2 RBD-targeting neutralizing antibody H014, nor the isotype control antibody (anti-H7N9) could prevent membrane fusion under similar conditions (Fig. 5B). Furthermore, we performed live SARS-CoV-2 neutralization assay in a post-binding manner in Huh7 cells. Briefly, Huh7 cells were infected with 100 PFU of SARS-CoV-2 for 1 h at 4°C. Unbound viral particles were washed away using buffer. After that, cells were further cultured in the presence of a series of concentrations (0, 4, 20 and 100 nM) of HB27, or 100 nM of H014 at 37°C for 48 h. Similar to S-mediated cell–cell fusion, the large syncytiums formed by live SARS-CoV-2 infected Huh7 cells were observed in the absence of HB27 and the presence of 100 nM H014 (Fig. 5C). Expectedly, HB27 could significantly inhibit SARS-CoV-2 mediated formation of the syncytiums in a dose-dependent fashion and completely block the cell–cell fusion at 100 nM (Fig. 5C). Notably, such inhibition, to some extent, can possibly be attributed to the ability of HB27 to strip SARS-CoV-2 off the cell surface. To further characterize the molecular basis for fusion inhibition by HB27, we established an *in vitro* membrane fusion assay in which treatments of purified SARS-CoV-2 virions by trypsin and ACE2 could trigger viral membrane fusion with liposome in an acidic environment. Liposome fusion results show that HB27, but not H014, is capable of efficiently blocking pH-dependent fusion of SARS-CoV-2 with liposomes in a dose-dependent manner (Fig. 5D). The blockage of membrane fusion by HB27 is likely another important mechanism of neutralization. However, given the relatively higher concentration of HB27 needed to block viral membrane fusion, blocking viral attachment to its host cell receptor is likely to be the main mechanism of neutralization.

## STRUCTURAL BASIS FOR THE SARS-CoV-2-SPECIFIC BINDING OF HB27

To delineate the structural basis for HB27-mediated specific neutralization, we determined the cryo-EM structure of a prefusion stabilized SARS-CoV-2 S ectodomain trimer in complex with the HB27 Fab fragment using single particle reconstruction. Similar to previously published studies on *apo* SARS-CoV-2 S trimer, two distinct conformational states referred to as the ‘closed’ and ‘open’ RBDs were observed in the structure of the complex (Fig. 6A). Cryo-EM characterization of the complex showed full occupancy with one Fab bound to each RBD of the homotrimeric S. We asymmetrically reconstructed the complex structure at an overall resolution of 3.5 Å, which represents two ‘open’ and one ‘closed’ RBDs (Fig. 6A, Supplementary Figs S3–S4 and Table S2). The initial maps for the binding interface between RBD and HB27 were relatively weak due to conformational heterogeneity, which is in line with the structural observations of stochastic RBD rotations at different angles while switching from the ‘closed’ to ‘open’ states (Fig. 6B and C). In order to improve the local resolution, we employed a ‘block-based’ reconstruction approach, which resulted in a 3.9 Å resolution, enabling reliable analysis of the interaction interface (Supplementary Figs S3 and S4).

**Figure 6.**
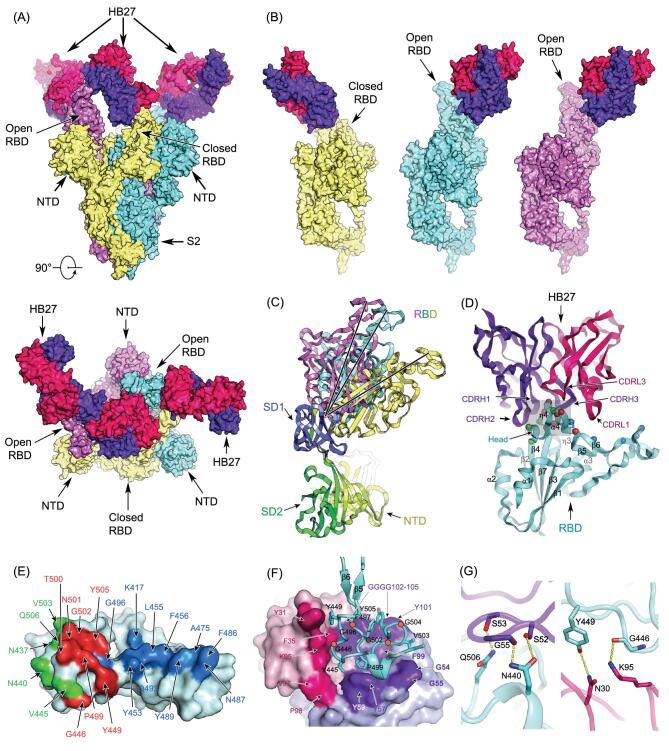
Structure and interaction of the SARS-CoV-2 S trimer with HB27. (A) Orthogonal views of SARS-CoV-2 S trimer in complex with three copies of HB27 Fab. (B) Individual views of the three monomers each complexed with one HB27 Fab. (A) and (B) The S trimer and HB27 are rendered as molecular surfaces. Three monomers of the S trimer are colored in yellow, cyan and violet, respectively. The HB27 light and heavy chains are colored in hotpink and purpleblue, respectively. RBD: receptor binding domain. NTD: N-terminal domain. S2: the S2 subunit. (C) S1 subunits of the three monomers from SARS-CoV-2 S trimer complexed with HB27 are superposed; HB27 Fabs are not shown. All domains are presented as ribbon diagrams. Three RBD domains are colored in yellow, cyan and violet, respectively. SD1: subdomain 1. SD2: subdomain 2. (D) Cartoon representations of the structure of SARS-CoV-2 RBD in complex with HB27. The RBD is cyan, and the light and heavy chains of HB27 are hotpink and purpleblue, respectively. Residues constituting the HB27 epitope and the RBM are drawn as spheres and colored in green and blue, respectively. The overlapped residues between the HB27 epitope and the RBM are colored in red. The CDRs involved in the interactions with the RBD are labeled. CDR: complementary determining region. RBM: receptor binding motif. (E) Residues in SARS-CoV-2 RBD comprising the HB27 epitope and RBM are labeled. The RBD is rendered with a cyan

**Figure 6.**
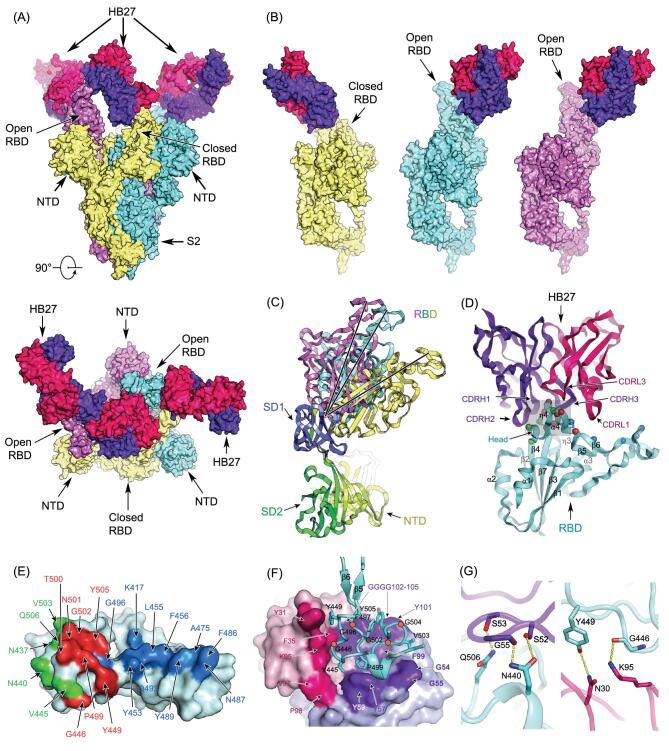
*Continued.* surface. Blue, green and red mark the HB27 epitope, the receptor-binding motif (RBM), and overlapped residues of them both, respectively. (F) Hydrophobic interactions between SARS-CoV-2 RBD and HB27. The RBD is shown as cyan ribbon diagrams, and the residues of which involved in hydrophobic interactions with HB27 are shown as side chains and labeled. The four dark orange circles mark the positions of four glycine residues. The HB27 light and heavy chain are rendered as light pink and pale blue molecular surfaces, respectively, of which the residues involved in the hydrophobic interactions with the RBD are highlighted in hotpink and purpleblue and labeled. (G) A few key interactions between SARS-CoV-2 RBD and the HB27 heavy (left) and light chain (right). Hydrogen bonds are presented as dashed lines.

HB27 binds to the apical head of RBD, partially overlapping with the edge of the receptor-binding motif (RBM) core. This binding was independent of glycan recognition (Fig. 6D and E). The head of RBD inserts into the cavity constructed by five complementarity-determining regions (CDRs, CDRL1, CDRL3 and CDRH1-3), involving extensive hydrophobic interactions (Fig. 6F). The heavy and light chains bury ∼500 Å^2^ and ∼210 Å^2^ of the surface area of the epitope, respectively. Tight binding is further facilitated by five hydrogen bonds (Fig. 6G and Supplementary Table S3). HB27 epitope includes 12 residues, of which only seven residues are conserved between SARS-CoV-2 and SARS-CoV, explaining its specificity for SARS-CoV-2 for binding and neutralization (Supplementary Fig. S5A). Although a number of point mutations in the RBD have been reported in currently circulating strains, none of these mutations lie within the HB27 epitope (Supplementary Fig. S5A). To test the spectrum of neutralizing activities of HB27 against currently circulating strains of SARS-CoV-2, RBD mutants bearing various amino acid substitutions reported were expressed and evaluated for their binding affinities to HB27. In line with structural analysis, all the RBD mutants exhibited comparable binding abilities (Supplementary Fig. S5B). More recently, SARS-CoV-2 isolates encoding a D614G mutation in the C-terminal region of the S1 predominate [35]. To investigate the neutralizing activities against this more contagious isolate, SARS-CoV-2 PSV harboring the D614G mutation was constructed. Compared to the wild type, HB27 showed similar binding affinities and neutralizing activities against the D614G mutant (Supplementary Fig. S6), indicating that HB27 possibly exhibits broad neutralization activity against SARS-CoV-2 strains currently circulating worldwide.

## STRUCTURAL DISSECTION OF THE MECHANISM OF NEUTRALIZATION OF SARS-CoV-2 BY HB27

**Figure 7.**
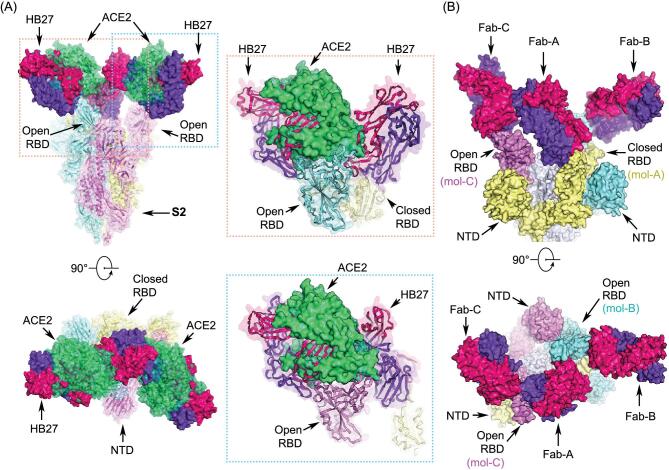
Structural basis for neutralization of SARS-CoV-2 by HB27. (A) Orthogonal views of the clashes between HB27 Fabs and ACE2 upon binding to SARS-CoV-2 S trimer. The SARS-CoV-2 S trimer is presented as ribbon diagrams and translucent molecular surfaces with three monomers colored in cyan, yellow and violet, respectively. The three copies of HB27 Fabs are rendered as molecular surfaces colored the same as in Fig. 6. The superposed ACE2 is presented as green ribbon diagrams as well as translucent molecular surface. Insets are close-up views of the clashes between ACE2 and HB27 upon binding to SARS-CoV-2 RBD. (B) Orthogonal views of the structure of HB27 Fab-A, Fab-B and Fab-C complexed with SARS-CoV-2 RBD. The S1 subunits of SARS-CoV-2 S trimer are rendered as cyan, yellow and violet surfaces and the S2 subunits are rendered as gray surfaces.

Results of our functional studies revealed that HB27 could completely block the interactions of SARS-CoV-2 with ACE2 (Fig. 4). To decipher the structural basis for this ability of HB27, the complex structures of SARS-CoV-2 trimer/HB27-Fab and SARS-CoV-2 RBD/ACE2 were superimposed. The superimposition of the structures revealed that HB27 could sterically hinder ACE2 binding (Fig. 7A). Out of the 12 residues in the HB27 epitope, seven residues are involved in tight contacts with ACE2 (Fig. 6E and Supplementary Fig. S7). In addition, the three HB27 Fabs act in synergy to abolish ACE2 binding, in which binding of any one ACE2 molecule is sterically hindered by two adjacent HB27 (Fig. 7A). Unlike most structural studies of the *apo* SARS-CoV-2 S trimer or complexes with a major configuration corresponding to one ‘open’ RBD and the other two RBDs in ‘closed’ states [11,13,16,29], only one conformational state with one ‘closed’ RBD (mol A) and two ‘open’ RBDs (mol B and C) was observed in our complex structure. Interestingly, Fab-A that binds the closed RBD lies between two open RBDs, forming contacts with the mol B-RBD and the Fab-C located in proximity to the mol C-RBD (Fig. 7B). Probably acting as a bridge, the Fab-A, to some extent, anchors links of all three RBDs and restrains their conformational transitions (Fig. 7B). Perhaps correlated with this, HB27 possesses the ability to disrupt the membrane fusion event through restraining the conformational changes playing out during the progression from the prefusion to the postfusion state. Collectively these data suggest that HB27 might prevent both the attachment of SARS-CoV-2 to host cells and viral fusion with endosomal membrane. However, fusion blockade by HB27 might be dependent on the uptakes of antibodies into the endosome.

## DISCUSSION

SARS-CoV-2 shares about 80% sequence identity with SARS-CoV, implying that both these viral strains share a similar mechanism of establishing an infection, including targeting a similar spectrum of host cells, employing a similar entry pathway and hijacking the same cellular receptor [1,5]. Both cross-reactive and virus-specific human NAbs have been identified, despite around 77% of amino-acid sequence identity between the S of SARS-CoV-2 and SARS-CoV [18,19,29,36–39]. It is important to decipher the immunogenic mechanism to discover patterns of different patches comprising different residues eliciting cross-reactive or virus-specific NAbs with various neutralization mechanisms. Currently, several cross-reactive mAbs, including CR3022, H014 and S309, screened from convalescent SARS patients or via immunization using SARS-CoV RBD, show distinct neutralizing activities against SARS-CoV-2 [18,29,36]. Structural analysis reveals that all these mAbs recognize conserved patches either distal from or proximal to the edge of the RBM, but not in the RBM. Interestingly, the corresponding epitope in both open and closed RBDs is accessible to S309, but accessible to H014 only in open RBDs, and can only be accessed by CR3022 when at least two RBDs are in the open conformation. The stoichiometric binding of Fab to the S trimer might correlate with the neutralizing activities, probably explaining the weak neutralization efficiency observed for CR3022. HB27 targets the less-conserved edge of the RBM core with a full occupancy for all RBDs. This structural observation supports the observed specificity of HB27 for SARS-CoV-2 and its highly potent neutralization of SARS-CoV-2.

Our results indicate that HB27 probably inhibits SARS-CoV-2 infection at multiple steps during the viral entry process. First, viral infection can be stalled by hindering the attachment of SARS-CoV-2 to host cells by preventing interactions between the RBD and ACE2, which is the major neutralization mechanism for most RBD-targeting NAbs. Upon virus attachment and entry into host cells, proteolytic activation at the S1/S2 boundary leads to S1 dissociation and a dramatic structural change in S2, which triggers viral membrane fusion [6]. To date, antibodies that are capable of interfering with coronavirus fusion have not been reported. HB27 may be involved in restraining the conformational changes required for the progression of the life cycle of the virus from the prefusion to the postfusion stage. Furthermore, recent studies suggest that the SARS-CoV-2 entry depends on ACE2 and cell surface protease TMPRSS2 [5,33]. A blockage of viral attachment to the host cell surface by HB27 possibly affects the colocalization of SARS-CoV-2 S with TMPRSS2 on the cell membrane. This may be yet another way employed by HB27 to prevent viral membrane fusion where the cleavage of S by TMPRSS2 is averted. Therefore, the potent neutralizing activity of HB27 probably results from its intervention at two steps of viral infection, locking away attachment of the virus to its receptor and blocking membrane fusion; resulting in a double lock.

Most importantly, the *in vivo* protection efficacy of HB27 was confirmed in two established mouse models. The results of these studies consistently showed that a single dose of HB27 either before or post SARS-CoV-2 exposure not only blocked viral replication in the lungs and trachea, but also prevented the pulmonary pathological damage. To date, only a few neutralizing antibodies have been tested in animal models [40,41]. Previously, we have shown that H014 reduced pulmonary viral loads by ∼100-fold in human ACE2 mice [29]. HB27 exhibits a more potent protective efficacy in reducing viral RNAs (∼11 000-fold) with a much lower administration dose (20 mg/kg vs. 50 mg/kg). The preliminary results on the efficacy of the antibody as well as the safety profile of HB27 in Rhesus macaques support testing of its potential in curing COVID-19 in clinical trials. In fact, while this manuscript was under preparation, HB27 entered clinical trials in China (registration number NCT04483375). More details can be found at https://clinicaltrials.gov/ct2/show/NCT04483375?cond=SCTA01&draw=2&rank=1.

In summary, our results not only show how increasing access to panels of authentic neutralizing monoclonal antibodies will facilitate structure-function studies to unpick the underlying biological processes of virus–host interactions, but also provide molecular basis for applying HB27 for potential COVID-19 treatment, highlighting the promise of antibody-based therapeutic interventions.

## Supplementary Material

nwaa297_Supplemental_File
