# Supplementary material for: Double lock of a potent human therapeutic monoclonal antibody against SARS-CoV-2
Source: Natl Sci Rev. 2020 Dec 18;8(3):nwaa297. doi: 10.1093/nsr/nwaa297 (PMC7798916; doi:10.1093/nsr/nwaa297)
Supplement: nwaa297_Supplemental_File [file nwaa297_supplemental_file.docx]

Supplemental Information for

**Double Lock of a Potent Human Neutralizing and Protective Monoclonal Antibody against SARS-CoV-2**

Ling Zhu^1,10^, Yong-Qiang Deng^2,10^, Rong-Rong Zhang^2,10^, Zhen Cui^1,10^,Chun-Yun Sun^3,10^, Chang-Fa Fan^4,10^, Xiaorui Xing^1,9,10^, Weijin Huang^5^, Qi Chen^2^, Na-Na Zhang^2^, Qing Ye^2^, Tian-Shu Cao^2^, Nan Wang^1^, Lei Wang^1^, Lei Cao^1^, Huiyu Wang^3^, Desheng Kong^3^, Juan Ma^3^, Chunxia Luo^3^, Yanjing Zhang^3^, Jianhui Nie^5^, Yao Sun^1^, Zhe Lv^1^, Neil Shaw^1^, Qianqian Li^5^, Xiao-Feng Li^2^, Junjie Hu^1^, Liangzhi Xie^3,6,7^*, Zihe Rao^1^*, Youchun Wang^5^*, Xiangxi Wang^1,8,^* and Cheng-Feng Qin^2^*

**This PDF file includes:**

Figures S1 to S7

Tables S1 to S3

**Methods**

**Facility and ethics**

Experiments involving live SARS-CoV-2 virus were performed in the enhanced biosafety level 3 (P3+) facilities in the Institute of Microbiology and Epidemiology, Academy of Military Medical Sciences. All animal experiments were approved by the Experimental Animal Committee of Laboratory Animal Center, AMMS (approval number: IACUC-DWZX-2020-001).

**Cells and viruses**

The human embryonic kidney 293T cell line (Cat: CRL-11268) used for pseudovirus (PSV) packaging was purchased from ATCC. Vero-E6 cells were purchased from Chinese Academy of Medical Sciences Cell Bank (Cat: GN017). Vero cells, 293T and Vero-E6 cells were grown in Dulbecco’s modified Eagle’s medium (DMEM) containing 10% (v/v) FBS. The SARS-CoV-2 viral strain BetaCoV/Beijing/IME-BJ01/2020 was originally isolated from a COVID-19 patient returning from Wuhan, China. The virus was amplified and titrated by standard plaque forming assay using Vero cells.

**Protein expression and purification**

Plasmids for protein expression were constructed by inserting the genomic sequences of SARS-CoV RBD (residues 306–527, GenBank: NC_004718.3), SARS-CoV-2 RBD (residues 319-541, GenBank: MN908947.3), and SARS-CoV-2 S trimer (residues 1–1208, GenBank:MN908947.3), respectively, into the mammalian expression vector pCAGGS with a C-terminal 2×StrepTag. Proline substitutions at residues 986 and 987, a “GSAS” instead of “RRAR” at the furin cleavage site were performed on the gene encoding S protein based on the research of Jason S. McLellan (1). Polyethylenimine was used to transiently transfect HEK Expi 293F cells (Thermo Fisher) with SARS-CoV RBD, SARS-CoV-2 RBD and SARS-CoV-2 S, respectively. StrepTactin resin (IBA) was used for protein purification from the cell supernatants, followed by size-exclusion chromatography with a Superose 6 10/300 column (GE Healthcare) or a Superdex 200 10/300 Increase column (GE Healthcare) in 20mM Tris, 200 mM NaCl, pH 8.0.

**Reagents, recombinant proteins and antibodies**

Recombinant RBD protein of SARS-CoV-2 with His tag (Cat: 40150-V08B2, monomer in solution), Recombinant ACE2 protein with His tag (Cat: 10108-H08H, monomer in solution), transfection reagent Sinofection (Cat: STF02), mammalian expression plasmids of full-length S protein with GFP tag at the C terminal (Cat: VG40589-ACGLN) were purchased from Sino Biological. Fetal bovine serum (FBS) (Cat: SA 112.02) were purchased from Lanzhou Minhai Bio-engineering. Luciferase assay system (Cat: E1501) was purchased from Promega. Anti-human IgG Fc/HRP (Cat: 5210-0165) were purchased from KPL. Goat anti-human IgG F(ab')2/HRP (Cat: 109-036-006) were purchased from Jackson ImmunoResearch.

**Generation of humanized anti-SARS-CoV-2 antibody HB27**

SARS-CoV-2 antibodies were screened from a phage-display scFv library constructed from the spleen mRNA of mice immunized with recombinant SARS-CoV-2 RBD protein. SARS-CoV-2 RBD was used as the bait to select for specific anti-RBD scFvs by biopanning and the scFvs exhibiting potent binding for SARS-CoV-2 RBD were generated as chimeric antibodies. The chimeric antibodies were expressed using HEK-293T transient transfection production system and examined for competition activities with ACE2 for binding to SARS-CoV-2 RBD and neutralizing activities against SARS-CoV-2 and SARS-CoV pseudoviruses. The chimeric antibody mhB27 exhibited high binding affinity to SARS-CoV-2 RBD and potent neutralizing activity against SARS-CoV-2 pseudoviruses, therefore its humanized version-HB27 (Fc modified IgG1 subtype) was further generated.

**Generation of Fab fragment**

The HB27 Fab fragment was prepared using Pierce FAB preparation Kit (Thermo Scientific) following the manufacturer’s instructions. In brief, following removal of the salt with a desalting column, the antibody was mixed with papain and incubated for digestion at 37 ˚C for 3-4 h. The HB27 Fab was separated using protein A affinity column and concentrated for further applications.

**Generation of mutant RBDs**

Genomic information of SARS-CoV-2 mutant strains were obtained from GISAID (https://platform.gisaid.org), selected site mutants within the RBD domain (residues 306-527) were conducted. The mutated RBD genes with His-tag were cloned into pSTEP2 vector and transfected into 293T cells for protein expression. Cell culture supernatants were collected and purified using IMAC resins.

**Protein-protein interaction identified by Octet**

Recombinant SARS-CoV-2 RBD-His was biotinylated and loaded onto SA sensor (Pall corporation), and then HB27 antibody or HB27 Fab fragments were added for real-time association and dissociation analysis using Octet96e (Fortebio). Data was processed with Data Analysis Octet.

**ELISA**

The competition between HB27 and ACE2 for binding to SARS-CoV-2 RBD, and the binding of HB27 antibody to mutant SARS-CoV-2 RBDs are examined by ELISA. Recombinant RBD protein was coated on 96-well plates using CBS buffer over night at 4℃. The plates were blocked in BSA at room temperature for 1 h. Recombinant ACE2 with an His-tag and serial diluted HB27 antibody were then added and incubated at room temperature for 1 h. After washing away the unbound proteins and antibodies, secondary antibody against His-tag with HRP labeling were added and incubated for 1 h before washed away. Developing buffer was added and incubated for 5-30 min, 1% H_2_SO_4_ was added to stop the reaction and absorbance at 450 nm was detected with a microplate reader. Recombinant RBD mutant proteins were coated on 96-well plates using PBS buffer at 4 °C for 12 h. After that BSA solution was used for blocking at 25 °C for 1 h. Serial diluted antibodies were then added and incubated at room temperature for 1 h. After washing away the unbound antibodies, secondary antibody against human IgG with HRP labeled was added and incubated for 1 h before washed away. For color development, TMB mixture solution was added and incubated for 5-30 min, then 1% H_2_SO_4_ was added to stop the reaction and absorbance at 450 nm was detected with a microplate reader.

**Flow cytometry**

HB27 was serial diluted and incubated with 293T-ACE2 cells or 293T-SARS-CoV-2-S cells together with recombinant SARS-CoV-2 RBD or ACE2 for 45 min, respectively. Following the washing away of unbound proteins, cells were incubated with FITC labeled secondary antibody for 20 min and subject to flow cytometer for examination of cellular binding. Data were analyzed using Flowjo and Graphpad.

**Production of pseudoviruses**

Pseudoviruses were prepared as previously described (2). In brief, 293T cells were transfected with the plasmids of SARS-CoV S or SARS-CoV-2 S, respectively. 24 hours later, transfected 293T cells were infected with VSV G pseudotyped virus (G*ΔG-VSV) at a multiplicity of infection (MOI) of 4. Two hours post infection, cells were washed three times using PBS, followed by adding complete culture medium. Twenty-four hours post infection, SARS-CoV or SARS-CoV-2 pseudoviruses were harvested, 0.45-μm filtered and stored at −80 °C.

**Pseudovirus neutralization assay**

Aliquots of a 100 μL of ~40,000 Vero-E6 cells/well were added into 96-well plates. 60 μL of SARS-CoV/ SARS-CoV-2 pseudoviruses and 60 μL of serial diluted antibody samples were incubated for 1 h at 37°C, after which the pseudovirus-mAb mixtures were added into the wells containing Vero-E6 cells. The 96-well plates were then incubated for 24 hours in a 5% CO_2_ environment at 37°C, then the luciferase luminescence (RLU) was measured using luciferase assay system following the manufacturer’s manual with a luminescence microplate reader. The neutralization percentage was calculated by the formula: Inhibition (%) = [1- (sample RLU- Blank RLU)/ (Positive Control RLU-Blank RLU)] (%). Neutralization titers of the antibodies were presented as 50% maximal inhibitory concentration (IC_50_).

**Immunofluorescence**

293T cells were transfected with SARS-CoV-2-S-GFP or ACE2-GFP. 48h later, cells were fixed with 4% paraformaldehyde (PFA) for 20 min at room temperature and stained for nuclei with 4,6-diamidino-2-phenylindole (DAPI). HB27 antibody was incubated for 1h, followed by incubation of RBD-His and anti-His-PE，or APC labelled ACE2-Fc for 20 min. The fluorescence images were recorded using a Nikon A1 confocal microscope.

**Liposome preparation**

Lipids, 1-palmitoyl-2-oleoyl-sn-glycero-3-phosphocholine (POPC; Avanti-Polar Lipids), 1,2-dioleoyl-sn-glycero-3-phospho-L-serine (DOPS; Avanti-Polar Lipids), 1,2-dihexadecanoyl- sn-glycero-3-phosphoethanolamine (Texas Red-DHPE; Sigma ChemicalCo.) were mixed in a 84.5:15:0.5 molar ratio and prepared as previously reported (3). The dried lipid film was hydrated at room temperature with 100 mM calcein (Sigma) in buffer (25 mM HEPES, 150 mM KCl, pH 7.4), and then the vesicles were extruded 25 times using the Mini-Extruder device (Avanti Polar Lipids) through Nuclepore filters (Whatman) with a pore size of 0.1 μm. Unincorporated calcein was separated from the liposomes using a Sephadex G-50 column. Liposomes (10 mM lipid on the basis of the input lipid) were stored at 4°C and used within 1 week.

**Liposome-binding and Calcein-leakage assays**

SARS-CoV-2 (~20 μg) was incubated with 0.1 μM trypsin (Sigma) at 37°C for 20 min. Then the virus was mixed with 0.3 μM ACE2 and HB27 antibody with the final concentration of 2.5 μM, 0.5 μM, 0.1 μM or 0.02 μM. The mixture was added to 0.1 mM liposomes in a total volume of 90 μl in a 96-well plate, and the fluorescence (excitation at 460 nm, emission at 509 nm) was monitored at 37 °C using a SpectraMax M5 Microplate Reader (Molecular Devices). At t = 0 sec, the pH of the medium were adjusted to 5.6 by addition of 10 μl of 1 M MES (morpholineethanesulfonic acid, pH 5.6) as *F*_0_. The emission fluorescence was recorded as *F*_t_ at 10 sec intervals. After 500 sec, 10 μl of 10% Triton X-100 was added to achieve complete release of the maximum fluorescence as *F*_100_. The fusion scale was calibrated such that 0% fusion corresponded to the initial excimer fluorescence value. The percentage of calcein leakage at each time point is defined as: leakage (%) = (*F*_t_ - *F*_0_) × 100 / (*F*_100_ - *F*_0_).

**Cell–cell fusion assay**

The establishment and detection of cell–cell fusion assay was performed as previously described (4). In brief, Vero-E6 cells were used as target cells and 293T cells transfected with SARS-CoV-2 S-GFP protein expression vectors were served as effector cells. Effector cells and target cells were co-cultured in the absence or presence of antibodies in DMEM containing 10% FBS for 48 h. After incubation, cells were fixed with 4% paraformaldehyde (PFA) at room temperature for 20 min and stained for nuclei with 4,6-diamidino-2-phenylindole (DAPI). The fluorescence images were recorded using a Leica SpeII confocal microscope. S-mediated cell-cell fusion was observed by the formation of multi-nucleated syncytia. Five fields were automatically collected in each well to count the number of fused and unfused cells and the antibody inhibition rate was calculated as following: fusion rate (FR) = (fused cell number) / (fused cell number+ unfused cell number), Inhibition%= (Positive Control (FR) –Sample (FR)) / (Positive Control (FR)) %. The experiment was performed three times.

**Negative stain**

Samples were diluted to a desired concentration (~0.02 mg/mL) and deposited onto freshly glow-discharged carbon-coated grids. After rinsing twice with deionized water, the grids were stained with 1% phosphotungstic acid (pH 7.0) and loaded onto a 120-kV transmission electron microscope (FEI) for inspection.

**Cryo-EM sample preparation and data collection**

HB27 Fab fragments and SARS-CoV-2 S ectodomain (1mg/ml) were purified and incubated at a ratio of 9 Fab molecules per S trimer. 3μL aliquots of the mixture were applied onto freshly glow-discharged C-flat R1.2/1.3 Cu grids. The grids were blotted for 3 s in 100% relative humidity for plunge-freezing (Vitrobot; FEI) in liquid ethane. The Cryo-EM data sets were collected at 300 kV with a Titan Krios microscope (Thermo Fisher) fitted with a Gatan K2 detector. Movies (32 frames, each 0.2 s, total dose 60 e^−^Å^−2^) were recorded at defocuses of between 1.25 and 2.7 μm using SerialEM, yielding a final pixel size of 1.05 Å.

**Image processing**

Micrographs of SARS-CoV-2 S trimer-HB27 Fab complex were recorded. The defocus values for each micrograph was determined using Gctf (5). Then particles were picked and extracted for 2D alignment and 3D classification by using the *apo* structure of SARS-CoV-2 S trimer (6) as the initial model in Relion (7). The best classes were selected and used for 3D refinement and postprocessing (estimate the B-factor automatically), yielding the final resolution of 3.5 Å based on the gold-standard Fourier shell correlation (threshold = 0.143) (8). However, the densities for the binding interface between RBD and HB27 are weak due to the conformational heterogeneity of the RBD. To solve this problems, we utilized the block-based reconstruction strategy (9-11) for focusing classification and refinement. Details on parameter settings can be found in structural determinations for the binding interface between RBD and H014(12) . In addition, local averaging of the RBD-Fab equivalent copies present in different classes further improves the resolution to 3.9 Å. All procedures were performed with Relion (7). The local resolution was evaluated by ResMap (13).

**Model building and refinement**

The structures of SARS-CoV-2 S trimer and a human Fab fragment (Protein Data Bank ID: 6VSB and 5N4J, respectively) were manually fitted into the refined map of SARS-CoV-2 S trimer-HB27 complex in Chimera (14) and then improved by manual real-space refinement in COOT (15). The atomic model was further subject to real-space positional and B-factor refinement using Phenix (16). The final models were evaluated using Molprobity (17). Detailed informatin of the data sets and refinement statistics are summarized in Table S2.

**Surface plasmon resonance**

The SARS-CoV-2 S trimer was immobilized onto a CM5 sensor to ~500 response units (RUs) using Biacore 8K (GE Healthcare). Serial diluted HB27 or Fab fragments or recombinant ACE2 flowed through the sensor. For competitive binding assays, the first sample was allowed to flow over the chip at a rate of 20 μl/min for 120 s, and then the second sample was injected at the same rate for another 120 s. The response units were recorded and analyzed.

**Plaque reduction neutralization tests (PRNT)**

The neutralization activity of HB27 against SARS-CoV-2 were examined by standard plaque reduction neutralization tests (PRNT) in Vero cells. In brief, 5-fold serial dilutions of HB27 were mixed with ~100 PFU of SARS-CoV-2 and incubated at 37 °C for 1 hour. The mixture was then added to Vero-E6 cell monolayers in a 12-well plate in duplicate and incubated at 37 °C for 1 hour. After which the mixture was removed, and 1 ml of 1.0% (w/v) LMP agarose (Promega) in DMEM supplemented with 4% (v/v) FBS was layered onto the infected cells. Following a two-day incubation at 37 °C, the wells were stained with 1% (w/v) crystal violet in 4% (v/v) formaldehyde for plaque visualization. The PRNT_50_ values were determined using non-linear regression analysis with GraphPad prism.

**Protection against SARS-CoV-2 challenge in hACE2 mice**

The *in vivo* protection efficacy of HB27 antibody was evaluated using a newly established mouse model based on a SARS-CoV-2 mouse adapted strain MASCp6 (18) and a humanized hACE2 mouse model (19), respectively. Briefly, a group of 6 to 8-week-old hACE2 humanized mice or BALB/c mice were intraperitoneally administrated with HB27 (20 mg/kg) before (prophylactic) and/or after (therapeutic) challenge with 5 × 10^5^ PFU of SARS-CoV-2 or 1.6×10^4^ PFU of MASCp6 via intranasal route, respectively. All mice were monitored daily for morbidity and mortality. The lung tissues of mice were collected at 3 and 5 dpi for viral RNA loads assay and HE staining.

**Viral RNA quantitation**

Viral RNA quantification was performed by RT-qPCR aplying One Step PrimeScript RT-PCR Kit (Takara, Japan). The primers and probe targeting against the gene of SARS-CoV-2 S used for RT-qPCR were CoV-F3 (5’-TCCTGGTGATTCTT CTTCAGGT-3’); CoV-R3 (5’-TCTGAGAGAGGGTCAAGTGC-3’); and CoV-P3 (5’- FAM-AGCTGCAGCACCAGCTGTCCA -BHQ1-3’), respectively.

**Pre- and post-adsorption inhibition assay**

Pre- and post-adsorption inhibition assays were performed as described previously (20). For the post-adsorption assay, SARS-CoV-2 was first added to Vero cells for 1 hour at 4 °C, and then the cells were washed three times, following which the mAb was added and incubated for 1 hour at 4 °C. For the pre-adsorption assay, the mAb was firstly incubated with SARS-CoV-2 for 1 hour at 4 °C before the mAb-virus mixture was added to Vero cells. After three washes using PBS, the PRNT was performed as described above. And the detection of the remaining amount of SARS-CoV-2 RNA on the surface of Vero cells after HB27 treatment was carried out with quantitative RT-PCR.

**Histology and Immunostaining**

Mouse tissues were excised and fixed with 10% neutral buffered formaline, and then dehydrated and embedded in paraffin. Sections of 4 μm thickness were obtained and stained with hematoxylin and eosin (H & E) following standard histological procedures. Images were recorded using Olympus BX51 microscope equipped with a DP72 camera.

**Toxicokinetics of HB27 in Rhesus Monkeys**

Rhesus macaques were randomly grouped into two groups, one group was given placebo and one group was given a single dose of HB27 at 500 mg/kg intravenously. Blood samples were collected at pre-dose, immediately after completion of dosing (± 1 minute), and 1h, 2h, 4 h, 8 h, 24 h (Day 2), 48 h (Day 3), 72(Day4), 120 h (Day 6), 168 h (Day 8) and 336 h (Day 15) after beginning of infusion. Serum concentration of HB27 was measured using ELISA.

**Clinical pathology of HB27 in Rhesus Monkeys**

Blood samples were collected via forelimb or hindlimb subcutaneous vein at predose and 3, 8 and 16 days postdose. Hematology parameters including white blood cells (WBC), lymphocytes (Lymph), red blood cells (RBC) and hemoglobin concentration (HGB) were measured using an ADVIA Hematology system. Clinical chemistry parameters including AST (aspartate transaminase), ALT (alanine transaminase) and ALP (alkaline phosphatase) and LDH (lactate dehydrogenase) were measured using TBA-120FR. BD FACS Calibur Flow Cytometry was used for determinations of CD4+, CD8+ T percentages.

**Data and materials availability**

Cryo-EM density maps have been deposited at the Electron Microscopy Data Bank with accession codes EMD-30503 (complex) and EMD-30500 (binding interface) and related atomic models has been deposited in the protein data bank under accession code 7CYP and 7CYH, respectively.

**Reference list:**

1. Wrapp, D, Wang, N, Corbett, KS*, et al.* Cryo-EM structure of the 2019-nCoV spike in the prefusion conformation. *Science*. 2020; **367**(6483): 1260-3.

2. Nie, J, Li, Q, Wu, J*, et al.* Establishment and validation of a pseudovirus neutralization assay for SARS-CoV-2. *Emerging microbes & infections*. 2020; **9**(1): 680-6.

3. Qiu, X, Lei, Y, Yang, P*, et al.* Structural basis for neutralization of Japanese encephalitis virus by two potent therapeutic antibodies. *Nature microbiology*. 2018; **3**(3): 287-94.

4. Xia, S, Liu, M, Wang, C*, et al.* Inhibition of SARS-CoV-2 (previously 2019-nCoV) infection by a highly potent pan-coronavirus fusion inhibitor targeting its spike protein that harbors a high capacity to mediate membrane fusion. *Cell research*. 2020; **30**(4): 343-55.

5. Zhang, K. Gctf: Real-time CTF determination and correction. *J Struct Biol*. 2016; **193**(1): 1-12.

6. Walls, AC, Park, YJ, Tortorici, MA*, et al.* Structure, Function, and Antigenicity of the SARS-CoV-2 Spike Glycoprotein. *Cell*. 2020; **181**(2): 281-92 e6.

7. Scheres, SH. Processing of Structurally Heterogeneous Cryo-EM Data in RELION. *Methods in enzymology*. 2016; **579**: 125-57.

8. Scheres, SH, Chen, S. Prevention of overfitting in cryo-EM structure determination. *Nat Methods*. 2012; **9**(9): 853-4.

9. Yang, Y, Yang, P, Wang, N*, et al.* Architecture of the herpesvirus genome-packaging complex and implications for DNA translocation. *Protein & cell*. 2020; **11**(5): 339-51.

10. Wang, N, Zhao, D, Wang, J*, et al.* Architecture of African swine fever virus and implications for viral assembly. *Science*. 2019; **366**(6465): 640-4.

11. Wang, N, Chen, W, Zhu, L*, et al.* Structures of the portal vertex reveal essential protein-protein interactions for Herpesvirus assembly and maturation. *Protein & cell*. 2020; **11**(5): 366-73.

12. Lv, Z, Deng, YQ, Ye, Q*, et al.* Structural basis for neutralization of SARS-CoV-2 and SARS-CoV by a potent therapeutic antibody. *Science (New York, NY)*. 2020; **369**(6510): 1505-9.

13. Kucukelbir, A, Sigworth, FJ, Tagare, HD. Quantifying the local resolution of cryo-EM density maps. *Nat Methods*. 2014; **11**(1): 63-5.

14. Pettersen, EF, Goddard, TD, Huang, CC*, et al.* UCSF Chimera--a visualization system for exploratory research and analysis. *J Comput Chem*. 2004; **25**(13): 1605-12.

15. Brown, A, Long, F, Nicholls, RA*, et al.* Tools for macromolecular model building and refinement into electron cryo-microscopy reconstructions. *Acta Crystallogr D Biol Crystallogr*. 2015; **71**(Pt 1): 136-53.

16. Afonine, PV, Grosse-Kunstleve, RW, Echols, N*, et al.* Towards automated crystallographic structure refinement with phenix.refine. *Acta Crystallogr D Biol Crystallogr*. 2012; **68**(Pt 4): 352-67.

17. Chen, VB, Arendall, WB, 3rd, Headd, JJ*, et al.* MolProbity: all-atom structure validation for macromolecular crystallography. *Acta Crystallogr D Biol Crystallogr*. 2010; **66**(Pt 1): 12-21.

18. Gu, H, Chen, Q, Yang, G*, et al.* Rapid adaptation of SARS-CoV-2 in BALB/c mice: Novel mouse model for vaccine efficacy. *bioRxiv*. 2020: 2020.05.02.073411.

19. Sun, SH, Chen, Q, Gu, HJ*, et al.* A Mouse Model of SARS-CoV-2 Infection and Pathogenesis. *Cell host & microbe*. 2020; **28**(1): 124-33 e4.

20. Wang, X, Zhu, L, Dang, M*, et al.* Potent neutralization of hepatitis A virus reveals a receptor mimic mechanism and the receptor recognition site. *Proc Natl Acad Sci U S A*. 2017; **114**(4): 770-5.

21. Kucukelbir, A, Sigworth, FJ, Tagare, HD. Quantifying the local resolution of cryo-EM density maps. *Nature methods*. 2014; **11**(1): 63-5.


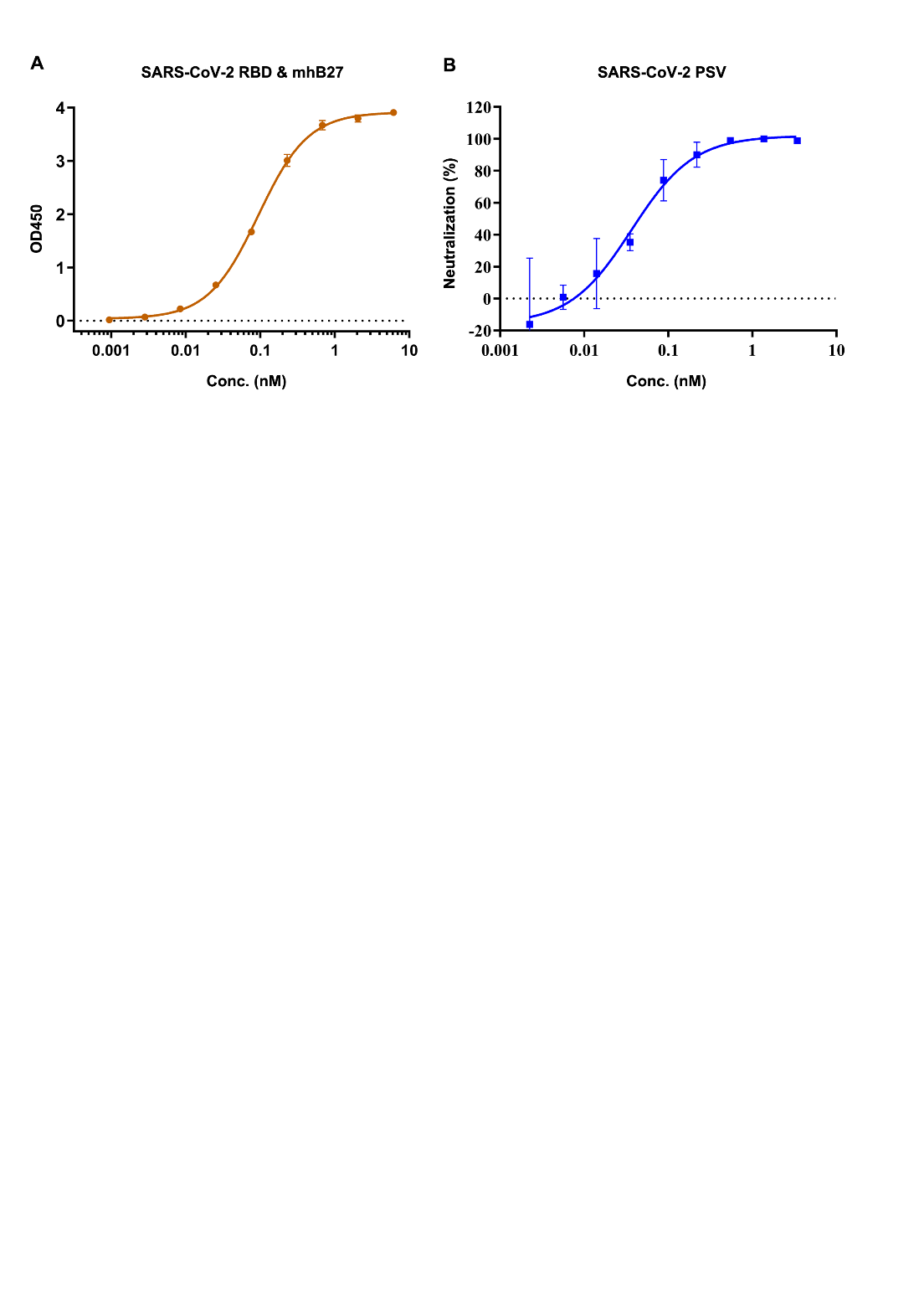


**Figure S1 Murine antibody mhB27 strongly binds SARS-CoV-2 RBD and neutralizes SARS-CoV-2 PSV.**

(A) Binding assay of mhB27 to SARS-CoV-2 RBD. mhB27 was serial diluted and tested its ability to bind to SARS-CoV-2 RBD by ELISA.

(B) Neutralizing activities of mhB27 against SARS-CoV-2 pseudoviruses (PSV).


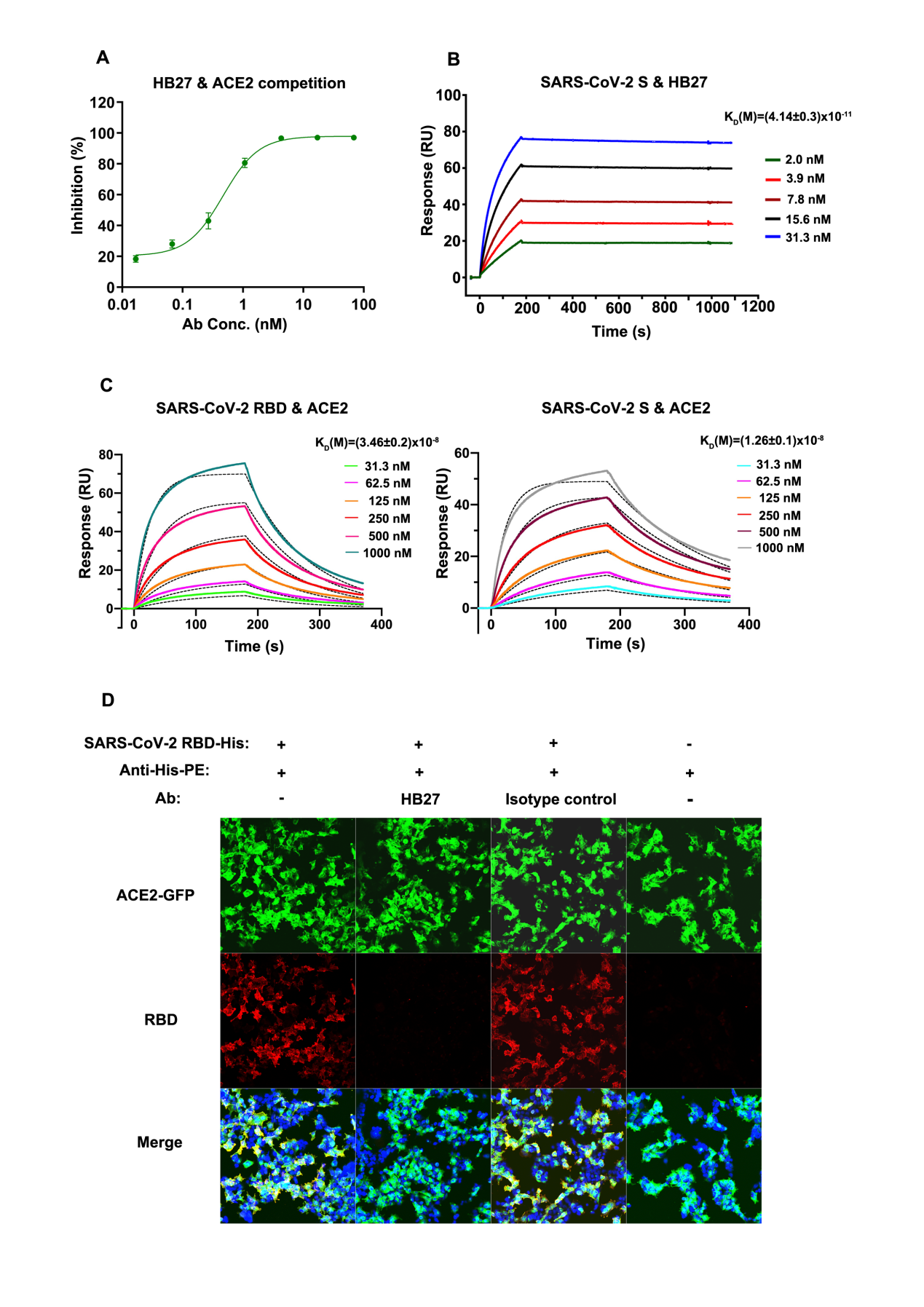


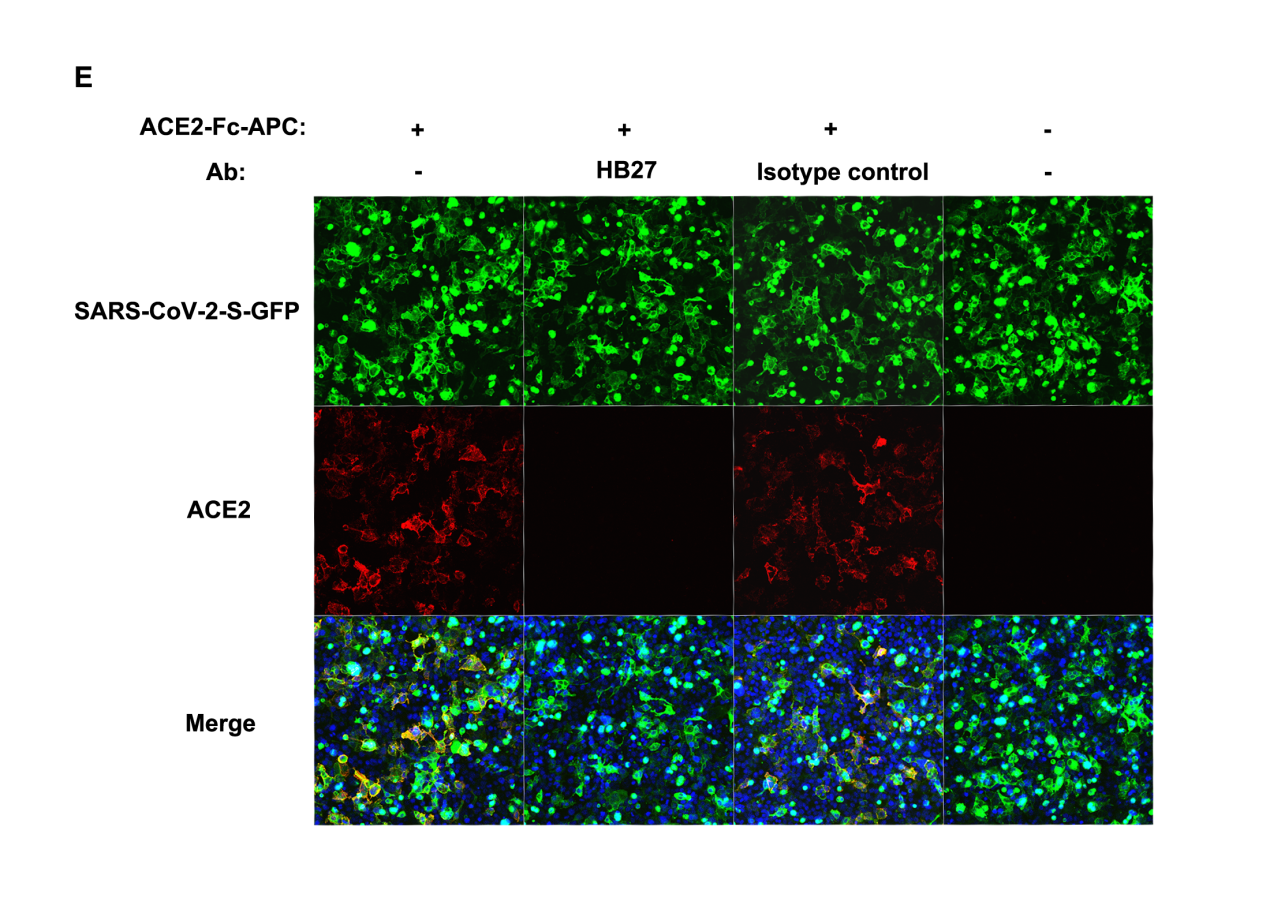


**Figure S2 HB27 potently competes with ACE2 for binding to SARS-CoV-2 RBD.**

(A) HB27 was demonstrated to compete with recombinant ACE2 for binding to SARS-CoV-2 RBD with an EC_50_ value of 0.5 nM by the enzyme-linked immunosorbent assay (ELISA).

(B) BIAcore SPR kinetic profile of SARS-CoV-2 S trimer and HB27. The binding affinity K_D_ (equilibrium dissociation constant, K_D_ = Kd/Ka, where Kd and Ka represent the dissociation rate constant and association rate constant, respectively) values were obtained using a series of HB27 concentrations and fitted in a global mode in each sensorgram.

(C) BIAcore SPR kinetic profiles of SARS-CoV-2 RBD (left panel) and S trimer (right panel) with ACE2. The binding affinity K_D_ (equilibrium dissociation constant, K_D_ = Kd/Ka, where Kd and Ka represent the dissociation rate constant and association rate constant, respectively) values were obtained using a series of HB27 concentrations and fitted in a global mode in each sensorgram.

(D) Competition of HB27 for SARS-CoV-2 RBD binding to 293T cells expressing GFP-tagged ACE2 as detected by immunofluorescence assay, scale bar, 100 μm. Anti-H7N9 mAb was used as an isotype control.

(E) Competition of HB27 for ACE2-Fc-Apc binding to 293T cells expressing GFP-tagged SARS-CoV-2-Spike as detected by immunofluorescence assay. Anti-H7N9 mAb was used as an isotype control.


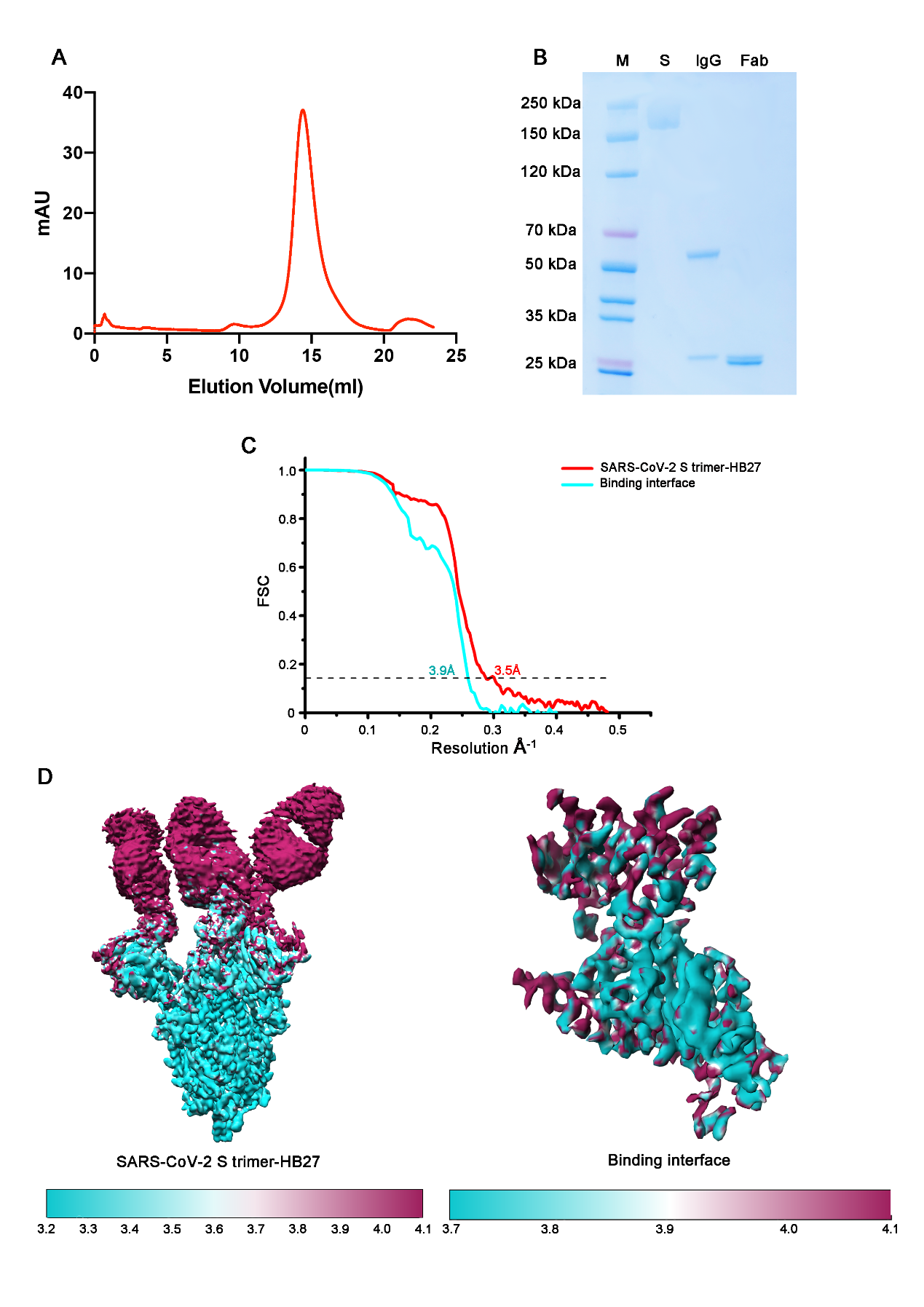


**
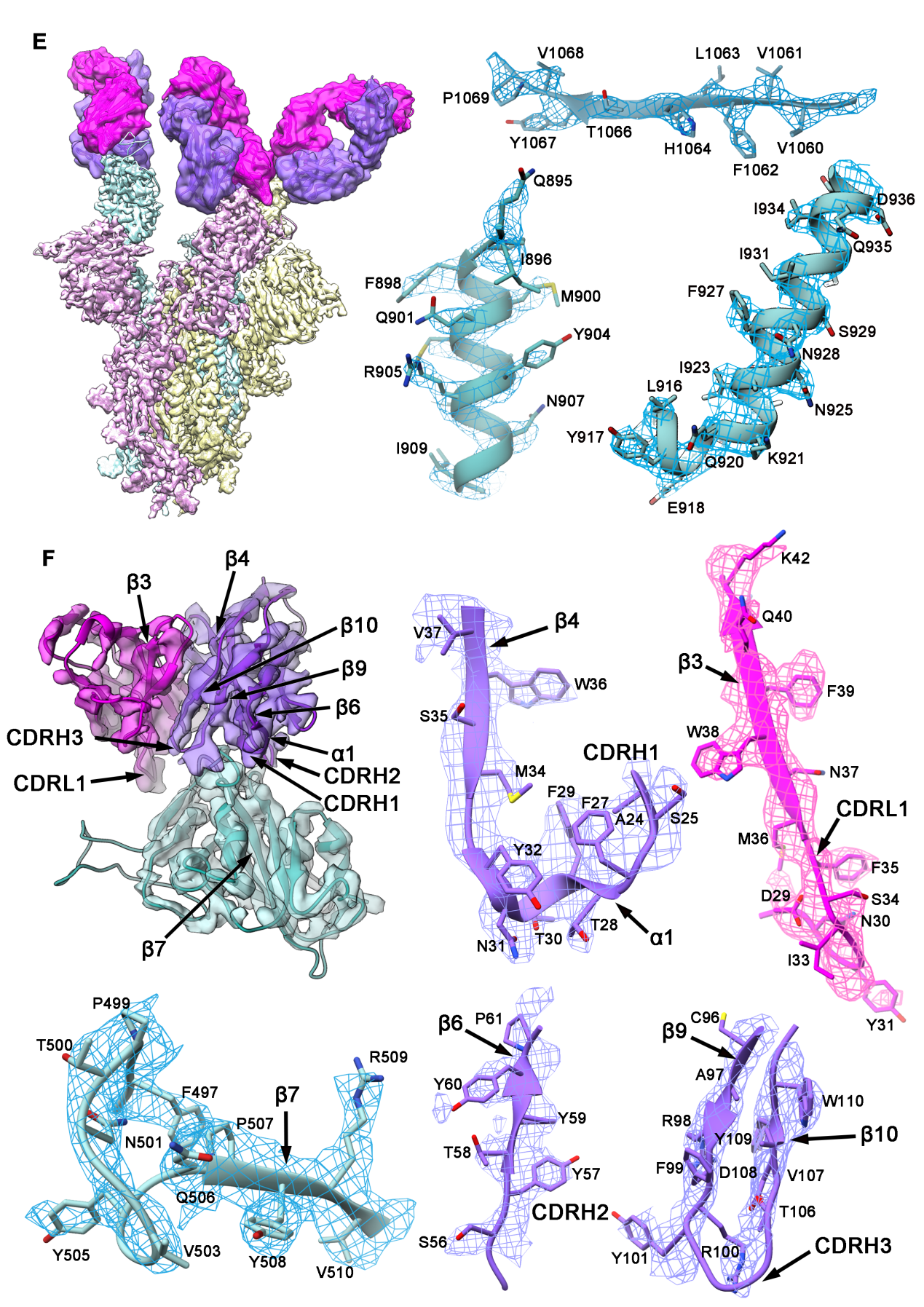
**

**Figure S3 Characterization of SARS-CoV-2 and HB27, and cryo-EM maps and atomic models of SARS-CoV-2 S and HB27 complex.**

(A) Gel filtration of SARS-CoV-2 S trimer.

(B) SDS-PAGE analysis of the SARS-CoV-2 S trimer, the HB27 IgG and the Fab fragment.

(C) The gold-standard Fourier Shell Correlation (FSC) curves of the final cryo-EM maps of the SARS-CoV-2 S trimer-HB27 Fabs complex and of the binding interface.

(D) Local resolution evaluations of the cryo-EM maps of SARS-CoV-2 S trimer complexed with three HB27 Fabs and the binding interface using ResMap (21) are shown.

(E) Cryo-EM map of SARS-CoV-2 S trimer complexed with three HB27 Fabs.

(F) Cryo-EM map of the binding interface between SARS-CoV-2 RBD and one HB27 Fab. The color scheme is the same as in Figure 6. The magnified panels illustrate both maps (mesh) and related atomic models. Residues are shown as sticks,

**
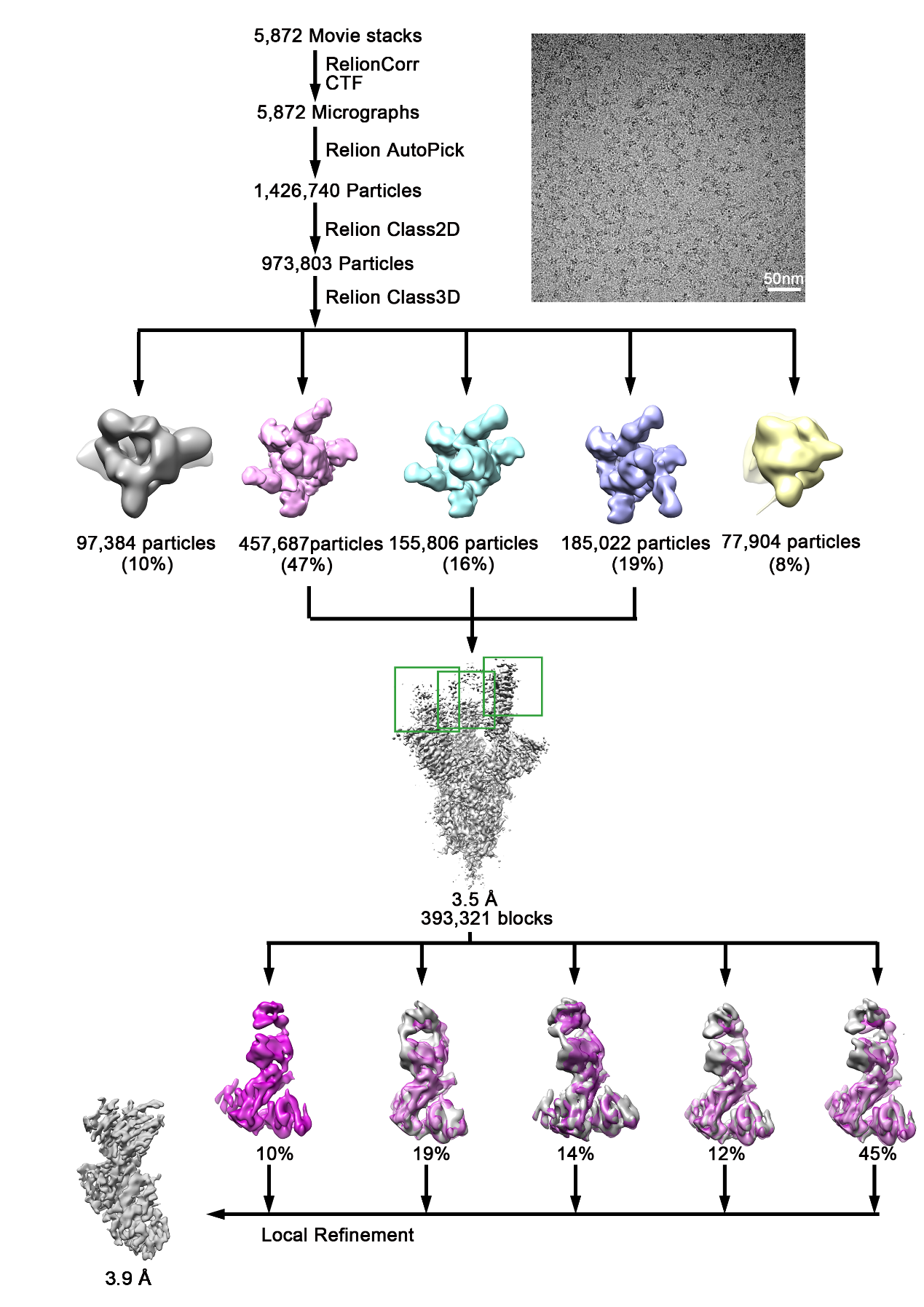
**

**Figure S4 Flowchart of Cryo-EM data processing of SARS-CoV-2 S trimer and HB27 complex.**


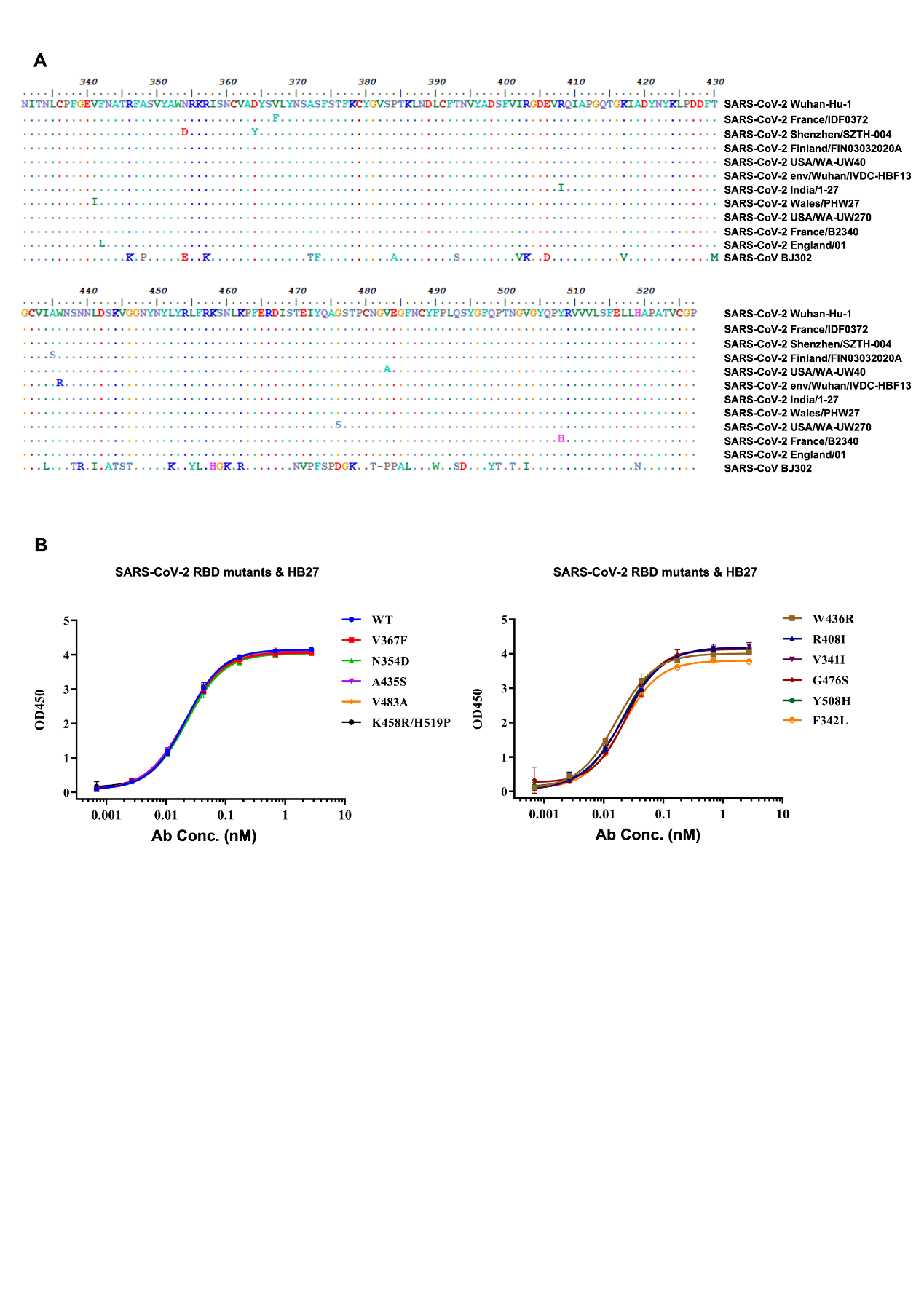
**Figure S5 HB27 strongly binds various SARS-CoV-2 RBD mutants.**

(A) Sequence alignments of the mutated RBDs of circulating SARS-CoV-2 strains used in (A) and SARS-CoV. The genome sequences used in the alignments were downloaded from NCBI and GISAID with accession numbers: NC_045512.2, EPI_ISL_406596, EPI_ISL_406595, EPI_ISL_413602, EPI_ISL_415605, EPI_ISL_408511, EPI_ISL_413522, EPI_ISL_415655, EPI_ISL_418055, EPI_ISL_416507, EPI_ISL_407071 and AY429078.1, respectively. The alignments were analyzed by Clustal W and BioEdit.

(B) ELISA binding assays of HB27 with selected SARS-CoV-2 RBD mutants. SARS-CoV-2 RBD proteins with previously reported site mutations were examined for their binding abilities to HB27.

**
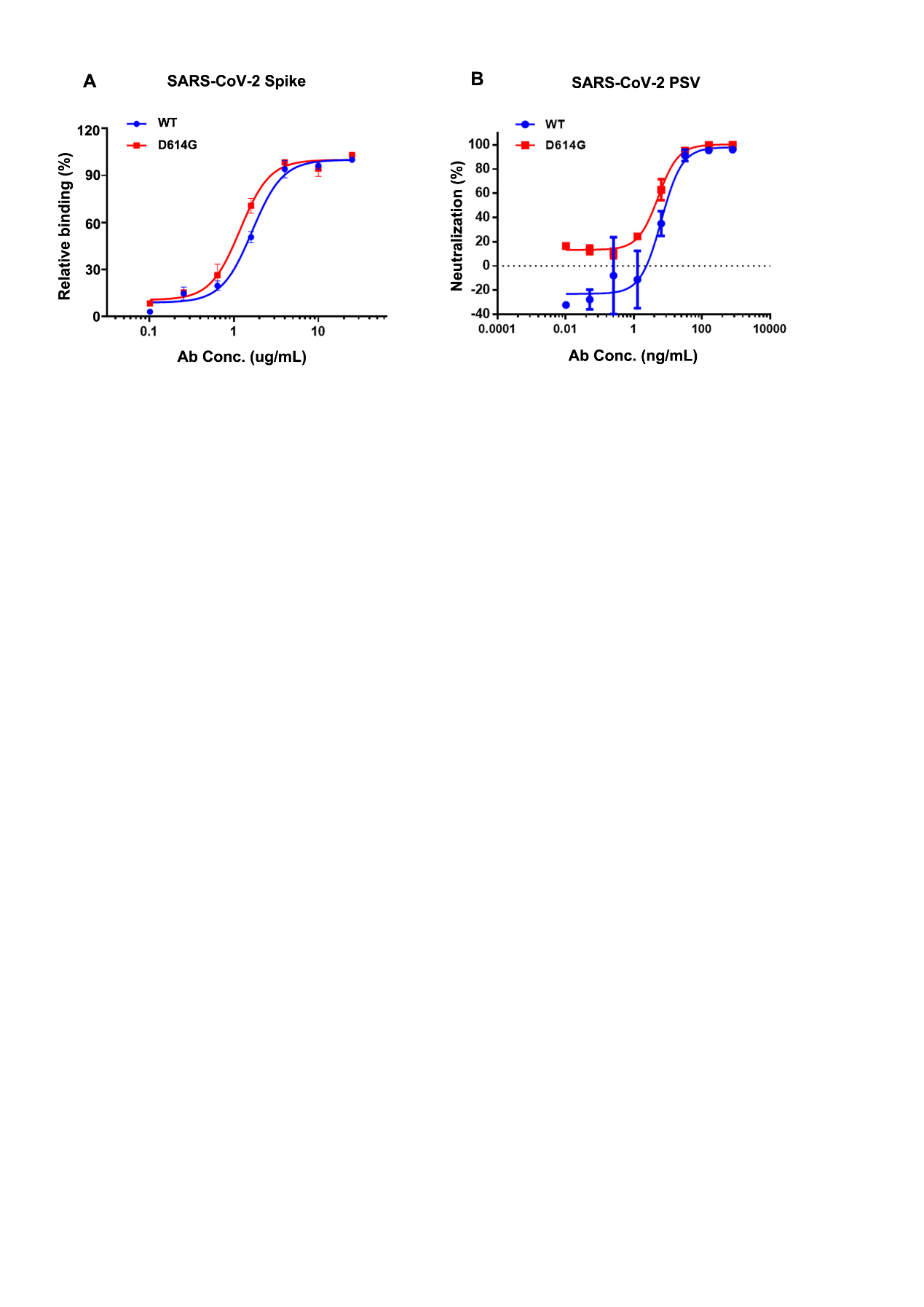
**

**Figure S6 HB27 potently binds and neutralizes SARS-CoV-2 wide type and mutant strain D614G.**

(A) The spike proteins of WT and D614G were transient expressed in 293T cells which were then examined for binding to HB27 by flow cytometry.

(B) Neutralizing activities of HB27 against SARS-CoV-2 WT and D614G pseudoviruses (PSV).

**
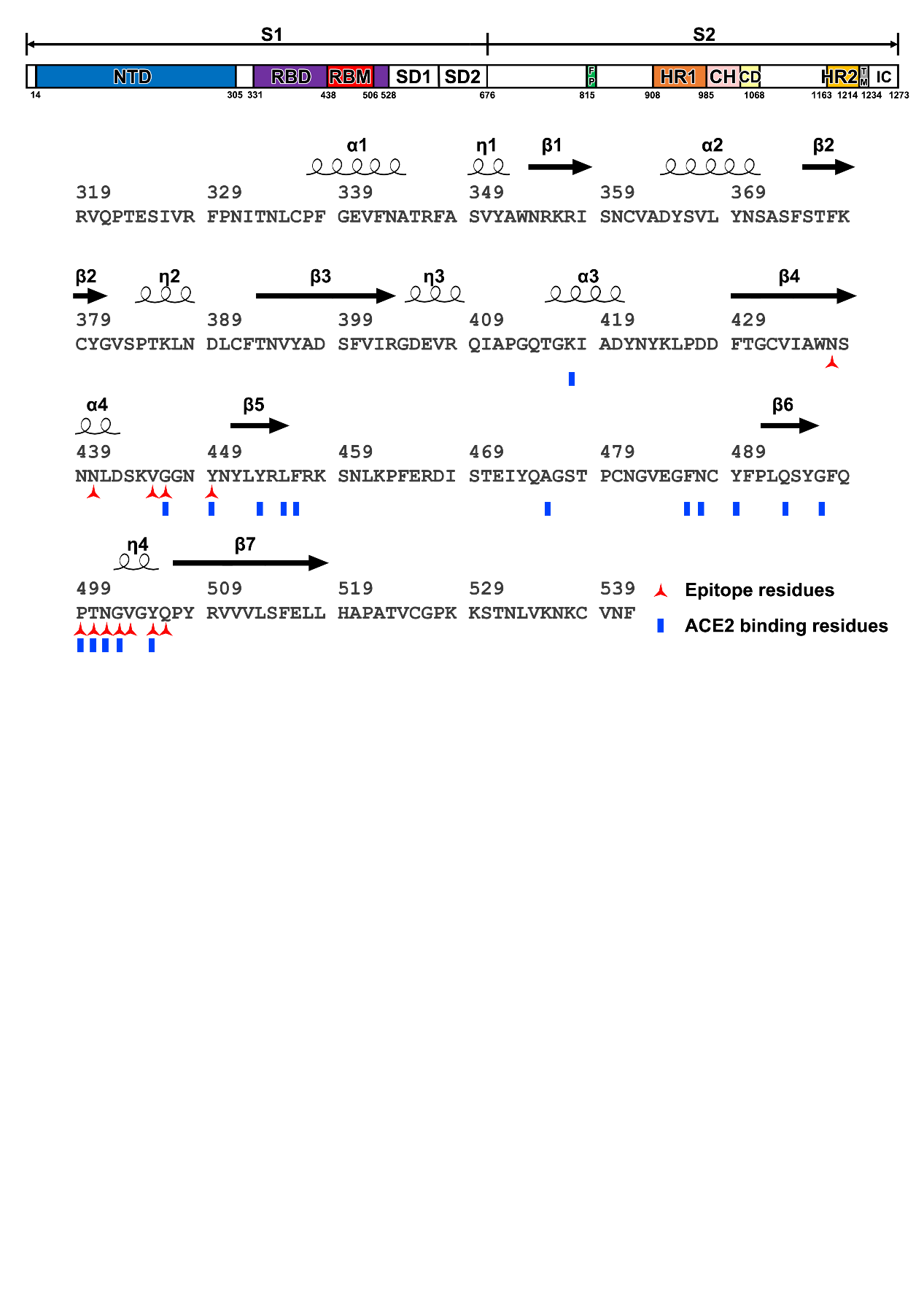
**

**Figure S7 Schematic diagram of SARS-CoV-2 S and the secondary structure of the RBD.**

(A) Overall topology of SARS-CoV-2 S. NTD: N-terminal domain; RBD: receptor-binding domain; RBM: receptor-binding motif; SD1: subdomain 1; SD2: subdomain 2; FP: fusion peptide; HR1: heptad repeat 1; HR2: heptad repeat 2; TM: transmembrane region; IC: intracellular domain.

(B) Protein sequence and the secondary structure of SARS-CoV-2 RBD. The red three-pointed stars and blue rectangles mark the residues in SARS-CoV-2 S RBD that interact with HB27 and ACE2, respectively.

**Table S1. Mean toxicokinetic parameters after intravenous injection of 500 mg/kg HB27 into Rhesus Monkey (0-336 h, n=2, mean ± SD).**

| **Sex** | **Parameters** | **t_1/2_** | **C_max_** | **AUC_last_** | **Vd** | **Cl** | **MRT** |
| --- | --- | --- | --- | --- | --- | --- | --- |
|  |  | **(h)** | **(mg/mL)** | **(h*mg/mL)** | **(mL/Kg)** | **(mL/h/Kg)** | **(h)** |
| **Male** | Mean | 278 | 12.8 | 1510 | 79.8 | 0.199 | 369 |
| **(n=2)** | SD | 27.3 | 1.41 | 71.9 | 5.02 | 0.00702 | 51.2 |
| **Female** | Mean | 201 | 12.9 | 1400 | 71.8 | 0.256 | 275 |
| **(n=2)** | SD | 45.8 | 0.212 | 233 | 4.5 | 0.0741 | 69.8 |

**Notes:**

t_1/2_: half time (or half life)

T_max_: time at maximum concentration

C_max_: maximum concentration

AUC_last_: area under the concentration-time curve from time zero to the last time point

Vd: volume of distribution

Cl: plasma clearance

MRT: mean residence time

**Table S2. Cryo-EM data collection and model reﬁnement statistics.**

| **Data collection and reconstruction statistics** | | |
| --- | --- | --- |
| Protein | SARS-CoV-2 S-HB27 | Binding interface |
| Voltage (kV) | 300 | 300 |
| Detector | K2 | K2 |
| Pixel size (Å) | 1.04 | 1.04 |
| Electron dose (e^-^/Å^2^) | 60 | 60 |
| Defocus range (μm) | 1.25-2.7 | 1.25-2.7 |
| Final particles | 798,515 | 393,321 |
| Final resolution (Å) | 3.5 | 3.9 |
| **Models refinement and validation statistics** | | |
| Ramachandran statistics | | |
| Favored (%) | 92.25 | 95.07 |
| Allowed (%) | 6.65 | 3.12 |
| Outliers (%) | 1.09 | 1.81 |
| Rotamer outliers (%) | 0.18 | 0.22 |
| R.m.s.d | | |
| Bond lengths (Å) | 0.012 | 0.014 |
| Bond angles (°) | 1.288 | 1.374 |

**Table S3. Residues of HB27 Fab interacting with the SARS-CoV-2 S trimer at the binding interface (d < 4 Å).**

| **S-RBD** | |  | **HB27 Fab** | | |
| --- | --- | --- | --- | --- | --- |
| **Location** | **Residues** |  | **Heavy chain** |  | **Light chain** |
| **β4** | N437 |  | G54, G55 |  |  |
| **α4** | N440 |  | S52, G55, S56, Y57 |  |  |
| **α4-β5** | V445 |  | Y57 |  |  |
|  | G446 |  |  |  | K95 |
|  | Y449 |  |  |  | N30, Y31 |
| **β6-η4** | P499 |  | Y57 |  |  |
|  | T500 |  | E50 |  |  |
|  | N501 |  | G102 |  |  |
| **η4** | G502 |  | N31, Y101 |  |  |
|  | V503 |  | N31, S53 |  |  |
|  | Y505 |  | Y101, G102 |  |  |
| **η4-β7** | Q506 |  | S53 |  |  |
